# Carotenoids, Fatty Acids, and Volatile Compounds in Apricot Cultivars from Romania—A Chemometric Approach

**DOI:** 10.3390/antiox9070562

**Published:** 2020-06-27

**Authors:** Adela Pintea, Francisc Vasile Dulf, Andrea Bunea, Sonia Ancuța Socaci, Elena Andreea Pop, Vlăduț-Alexandru Opriță, Daniele Giuffrida, Francesco Cacciola, Giovanni Bartolomeo, Luigi Mondello

**Affiliations:** 1 Department of Chemistry and Biochemistry, University of Agricultural Sciences and Veterinary Medicine Cluj-Napoca, 400372 Cluj-Napoca, Romania; apintea@usamvcluj.ro (A.P.); francisc.dulf@usamvcluj.ro (F.V.D.); andrea.bunea@usamvcluj.ro (A.B.); sonia.socaci@usamvcluj.ro (S.A.S.); andreea.e.pop@gmail.com (E.A.P.); 2 Research Station for Fruit Growing (RSFG), 907300 Valu lui Traian, Romania; olaviani@yahoo.co.uk; 3 Department of Biomedical, Dental, Morphological and Functional Imaging Sciences, University of Messina, 98168 Messina, Italy; dgiuffrida@unime.it (D.G.); gbartolomeo@unime.it (G.B.); 4 Department of Chemical, Biological, Pharmaceutical and Environmental Sciences, University of Messina, 98168 Messina, Italy; lmondello@unime.it; 5 Chromaleont s.r.l., c/o Department of Chemical, Biological, Pharmaceutical and Environmental Sciences, University of Messina, 98168 Messina, Italy; 6 Department of Sciences and Technologies for Human and Environment, University Campus Bio-Medico of Rome, 00125 Rome, Italy; 7 BeSep s.r.l., c/o Department of Chemical, Biological, Pharmaceutical and Environmental Sciences, University of Messina, 98168 Messina, Italy

**Keywords:** *Prunus armeniaca* L., carotenoids, fatty acids, volatiles

## Abstract

Lipophilic constituents are important for the color and aroma of apricots, but also for their health benefits. In the present study, carotenoids, fatty acids, and volatiles were analyzed in 11 apricot cultivars, from which nine were obtained in Romania. High performance liquid chromatography coupled to a diode array detector with atmospheric pressure chemical ionization and mass spectrometry (HPLC-DAD-APCI-MS methodology applied on unsaponified carotenoid extracts allowed the identification and quantification of 19 compounds. The predominant carotenoids in all cultivars were all*-trans*-β-carotene and its *cis* isomers. Lutein was present exclusively in non-esterified form, while β-cryptoxanthin was predominantly esterified, mainly with oleic, palmitic, lauric, and stearic acid. Moreover, β-cryptoxanthin linoleate, linolenate, and stearate were detected for the first time in Harogem cultivar. Variation in carotenoid content and composition was observed, with the highest carotenoid content being recorded in Tudor, Harogem, and Mamaia cultivars. The predominant fatty acids determined by gas chromatography–mass spectrometry (GC-MS) were linoleic (up to 47%), palmitic (up to 32.7%), and linolenic (up to 17.16%), with small variations among cultivars. In-tube extraction technique (ITEX)/GC-MS was applied for profiling the volatiles in apricot fruits and 120 compounds were identified, with terpenoids and esters as the most abundant classes. Principal component analysis (PCA) revealed that the carotenoids and the fatty acids profile can be used for variety authentication and discrimination in apricots.

## 1. Introduction

Apricots (*Prunus armeniaca* L.) are among the most consumed fruits worldwide—fresh, dried, as nectar, canned or jam. The world production in 2018 was estimated at 3,838,523 t with Turkey and Uzbekistan as the largest producers [1]. Romania produced more than 35,000 t of apricots in 2018 and is one of the leading European countries in apricot breeding, with a collection of more than 630 genotypes originating from different geographical areas in the world. In the frame of a large apricot improvement program which started in 1971, more than 40 new cultivars have been obtained by fruit research institutes [1,2].

Apricots are highly appreciated fruits due to their pleasant taste, flavor, and bright orange color of both the pulp and the peel. On average, the major constituents of apricot fruits are carbohydrates (6.8 g/100 g fresh weight (FW) from which 1.5 g fibers), proteins (0.4 g/ 100 g FW), and lipids (0.1 g/100 g FW) [3]. Saccharose, glucose, and fructose are the most important soluble sugars, while citric and malic acids are the major organic acids, with concentrations strongly affected by the cultivar and the stage of maturity [4]. A higher concentration of sorbitol (up to 26.84 mg/100 g dry weight (DW)) was found in Malatya apricots compared to other varieties [5]. In addition, it was proven that the composition and the ration of sugars in the hemicelluloses fraction can be used for the differentiation and authentication of fruit species (and derived products) of the genus *Prunus armeniaca* L. and *Prunus persica* L. [6]. Among the secondary metabolites, polyphenols and carotenoids are the most important representatives. A total polyphenol content ranging from 32.6 to 160.0 mg/100 g FW was reported in Spanish cultivars [7] and 0.3–7.4 mg gallic acid equivalent/g FW in apricot cultivars of Greek and American origin [8]. The major phenolic compounds in apricots are procyanidins, hydroxycinnamic acid derivatives, flavonols, and anthocyanins; all of which are more concentrated in the peel than in the flesh [7,9,10]. Epicatechin and procyanidin B2 are of special interest for detecting the adulteration, as they are present in apricot purees but absent in the least expensive peach puree [11]. Red skin apricot cultivars accumulated up to 296 mg/kg anthocyanins during ripening, with cyanidin-3-*O*-rutinoside representing the major compound [12]. However, the bright orange color of apricot flesh and skin is mainly due to the presence of carotenoids. According to the criteria proposed by Britton and Khachik [13], apricots are considered fruits with high to very high (>2 mg/100 g of FW) β-carotene content. Strongly influenced by the cultivar, geographical origin, and stage of ripening, the total carotenoid content in apricot ranges from 14.83–91.89 mg/100 g of DW in cultivars of Malatya apricots [5]; 9.5–37.8 μg/g FW in Greek and American cultivars [8]; 21–55 mg/kg FW in Hungarian varieties [14]; 2196 μg/100 g FW [15]; 1512–16500 μg/100 g FW in Spanish cultivars [7]. β-carotene (all*-trans*) represents more than 60% of the total carotenoids in all the varieties analyzed so far, followed by its *cis* isomers. Depending on the cultivar, α-carotene, γ-carotene, phtytoene, phytofluene, β-cryptoxanthin (free and esterified), lutein, and zeaxanthin were also identified [15,16,17,18]. Vitamin C (ascorbic acid) is a major contributor to the nutritional and antioxidant properties of fruits, with concentrations between 20.6 and 96.8 mg/100 g DW in Turkish Malatya apricots, significantly different depending on the type of cultivar [5].

Apricots are good sources of minerals, with potassium as the most important ion (1.5–1.7%), followed by calcium (0.06–0.26%) and magnesium (0.04–0.075%) [8,19]. Moreover, a daily consumption of 3–4 fresh apricots may supply the daily requirement of K, Mg, Fe, Se, and Mn and half of P and Zn for an average adult [5].

Although aroma compounds are minor in terms of weight, they are extremely important for the taste and consumers’ acceptance, together with sugars and organic acids. A wide range of volatile compounds have been identified in apricots cultivars, which belong to aldehydes, alcohols, esters, terpenes, and lactones [4,20]. 

As apricots have a very short shelf life, more than 40% of total production is processed by drying (sun, hot air or microwave), freezing or canning [21]. Carotenoids seems to be better retained in fruits than polyphenols after both industrial canning and domestic cooking, but they decrease during storage [22]. In addition to their nutritional value, apricots are associated with health benefits such as antioxidant, antimicrobial, anti-inflammatory, anti-hypertensive, anti-tumor, and anti-amyloidogenic effects [3,23]. 

Numerous studies demonstrated the large variability of the chemical composition of apricots, depending on the cultivar and geographical origin. Here we aimed to investigate the composition in lipophilic compounds of nine Romanian cultivars in comparison with two international cultivars, grown in similar conditions. The carotenoids, fatty acids, and volatiles composition of 11 different cultivars of *Prunus armeniaca*, from which nine were not previously characterized, were directly investigated by high performance liquid chromatography coupled to a diode array detector with atmospheric pressure chemical ionization and mass spectrometry (HPLC-DAD-APCI-MS) and gas chromatography–mass spectrometry (GC-MS) methodology. Crude unsaponified extracts were used in order to reveal the native composition of carotenoids in apricot samples. Furthermore, principal component analysis (PCA) was applied to reveal whether free or esterified carotenoids, fatty acids or volatile compounds can be used as markers for taxonomic classification and authenticity recognition.

## 2. Materials and Methods

### 2.1. Chemicals

Reagents and solvents used in extractions and sample preparation were of analytical, HPLC or LC/MS grade and were purchased from Merck Life Science (Merck KGaA, Darmstadt, Germany). Carotenoid standards, β-carotene, lycopene, β-cryptoxanthin, lutein, and zeaxanthin were purchased from Extrasynthese (Genay, France). The fatty acid methyl ester (FAME) standard (37 component FAME Mix, SUPELCO, catalog No: 47885-U) was purchased from Supelco Inc. (Bellefonte, PA, USA). 

### 2.2. Samples

Apricot cultivars Harogem (A1), Comandor (A2), Sirena (A3), Excelsior (A4), Sulina (A7), Olimp (A8), Mamaia (10), and Tudor (A11) were provided by the Research Station for Fruit Growing Constanța (38°29′ lat. and 44°10′ long.); Sulmona (A5), Umberto (A6), and Băneasa Red (A9) cultivars were provided by Domeniile Ostrov (44°03′ lat. and 28°17′ long). Except for A1 (CA) and A6 (IT), all the other cultivars were created by Fruit Research Stations in Romania: A2, A4, A8, A9, A10 (Fruit Growing Research Station Bǎneasa-Bucureşti); A3, A5, A7 (Station of Agricultural Research-Development Mǎrculeşti); and A11 (Research Station for Fruit Growing Constanţa), between 1979 and 2002. Regarding the color, A4 and A6 have yellow flesh and skin; A2, A5, and A7 have yellow-orange flesh and skin with red spots; A1, A3, A8, A9, A10, and A11 have orange flesh and orange-reddish skin. A11 is a medium ripening cultivar (end of June–first decade of July); A2, A4, A9, and A10 are medium-late ripening cultivars (second–third decade of July); A1, A3, A4, A6, and A7 are late ripening cultivars (first–third decade of August). Both farms are located in the Constanța County, southeast of Romania, between the Danube River and the Black Sea. This region has a temperate-continental climate with an average yearly temperature of 10.7 °C, characterized by mild winters with the lowest temperatures rarely descending below −17 °C and warm and very arid summers (200–250 mm during the active period [2]. The fruits were collected between 25 June–15 August 2014, when fully ripe according to their color and firmness. For each variety, three replicates of 10 fruits were harvested in three different field areas. Samples were stored at –20 °C until they were analyzed. 

### 2.3. Carotenoid Extraction

The carotenoids were extracted from fruit homogenates (20 g) with a mixture of methanol/ethyl acetate/petroleum ether (1:1:1, *v/v/v*) to color exhaustion. The combined extracts were filtered and then partitioned in a separation funnel with diethyl ether and saturated NaCl solution. The ether phase containing the pigments was filtered through anhydrous sodium sulphate to remove water and evaporated to dryness at 35 °C under vacuum, using a rotatory evaporator. Samples were stored at −20 °C until they were analyzed. The residue was dissolved in methyl tert-butyl ether (MTBE)/methanol (1:1, *v/v*) and filtered through 0.45 μm PTFE (polytetrafluoroethylene) filters and analyzed by HPLC. All experiments were performed under subdued light.

### 2.4. HPLC-DAD-MS Analysis of Carotenoids

The analyses were carried out on a Shimadzu Prominence LC-20A system (Shimadzu, Kyoto, Japan) equipped with a CBM-20A controller, two LC-20AD pumps, a DGU-20A_3_ degasser_,_ a manual injection valve (Rheodyne, Rohnert Park, CA, USA), and a SPD-M20A photo diode array detector. The data were processed with the software Shimadzu Labsolution ver. 5.53. For MS analyses, a mass spectrometer was used (LCMS-2020, Shimadzu, Kyoto, Japan), equipped with an atmospheric pressure chemical ionization (APCI) interface, both in positive and negative ionization modes. HPLC separations were performed on a C30 YMC column (250 × 4.6 mm; 3 µm particles); the mobile phases consisted of methanol/MTBE/water (86:12:2, *v/v/v*; eluent A) and methanol/MTBE/water (8:90:2, *v/v/v*; eluent B), using a gradient program as follows: 0 min, 0% B; 20 min, 0% B; 140 min, 100% B. The flow rate was 1 mL/min and the injection volume was 20 µL. The UV–Vis spectra were acquired in the range of 250–600 nm, while the chromatograms were extracted at 450 nm (sampling frequency: 12.5 Hz; time constant: 0.025 s). The MS was set as follows: scan, both APCI positive (+) and negative (−); nebulizing gas flow (N_2_): 4 L/min; event time: 0.25 s; detector voltage: 0.8 kV; m/z range: 350–1200; scan speed: 3750 m/s; interface voltage: ±4.5 kV; interface temperature: 350 °C; DL voltage: 0 V; DL temperature: 300 °C; heat block: 300 °C; Q-array: 0.0 V; RF: 90 V; sampling: 2 Hz. 

Quantitative analysis (HPLC-DAD) was performed using an identical equipment and mobile phase with those previously mentioned, but on a C30 YMC column (250 × 4.6 mm; 5 µm particles) and using external calibration with beta-carotene and beta-cryptoxanthin (1–100 μg/mL).

### 2.5. Lipid Extraction and Fatty Acids Analysis

Total lipids were extracted from 5 g of fresh fruit samples using chloroform/methanol (2:1, *v/v*) and quantified gravimetrically, as previously described [24,25]. Fatty acid methyl esters (FAMEs) were obtained from total lipids using the acid-catalyzed transesterification procedure described by Christie [26].

The analysis of FAMEs was achieved by gas chromatography–mass spectrometry (GC-MS) using a PerkinElmer Clarus 600 T GC-MS (PerkinElmer, Inc., Shelton, CT, USA) equipped with a Supelcowax 10 (60 m × 0.25 mm i.d.; 0.25 μm film thickness; Supelco Inc., Bellefonte, PA, USA) capillary column. Helium was used as the carrier gas at 0.8 mL/min. [25]. The following temperature program was used: 140 °C (hold 2 min); increase by 7 °C/min to 220 °C (hold 23 min). Injection (0.5 μL sample) was performed at a split ratio of 1: 24. The injector temperature was set to 210 °C. The MS working conditions were as follows: electron impact ionization voltage 70 eV (EI, positive ion electron impact mode), trap current of 100 μA, ion source temperature 150 °C, scan rate 0.14 scan/s, and scanned mass range 22–395 *m/z*.

The fatty acids were identified by comparison of both retention time and MS of the unknown peak to those of known standards and with data provided by MS database (NIST MS Search 2.0). The compositions of fatty acids in studied lipids were expressed as percentages (%) of the total FAME peak areas. All experiments were performed in triplicate. Statistical differences among samples were estimated using ANOVA (repeated measures ANOVA; Tukey’s multiple comparison test; GraphPad Prism Version 7.0, Graph Pad Software Inc., San Diego, CA, USA). A value of *p* < 0.05 was considered as statistically significant.

### 2.6. Volatile Analysis

The extraction of volatile compounds was performed using the in-tube extraction technique (ITEX) as described in our previous work [27] using a headspace vial in which 5 g of sample were mixed with 0.5 g NaCl and 2 mL H_2_O. The sealed vial was incubated at 60 °C for 40 min under continuous agitation. The volatile compounds were adsorbed on a Tenax fiber (ITEX-2 TrapTXTA, Tenax TA 80/100 mesh, ea) performing 40 extraction strokes and afterwards thermally desorbed directly into the GC-MS injector. The analysis of volatile compounds was carried out on a GC-MS QP-2010 (Shimadzu Scientific Instruments, Kyoto, Japan) model gas chromatograph–mass spectrometer equipped with a CombiPAL AOC5000 autosampler (CTC Analytics, Zwingen, Switzerland). The volatile compounds were separated on a Zebron ZB-5ms capillary column of 30 m × 0.25 mm i.d and 0.25 μm film thickness (Phenomenex, Torrance, CA, USA). The carrier gas was helium with a flow rate of 1.5 mL/min and split ratio of 1:2. The oven temperature program was as follows: start at 40 °C (hold for 5 min) to 120 °C with a 2 °C/min rate and hold for 2 min. Then, temperature was increased by 10 °C/min until 220 °C, where it was held for 5 min. 

The injector, ion-source, and interface temperatures were set at 250 °C. The MS detector was operating in electron impact (EI) ionization, scanning a mass range between 20 and 350 *m/z*. The tentative identification of the separated volatile compounds was achieved based on their mass spectra using the NIST27 and NIST147 mass spectra libraries. The identification process was also backed up by the compounds retention indices drawn from www.pherobase.com or www.flavornet.org. The concentration of each compound is expressed as relative percentage of total peaks area.

### 2.7. Statistical Analysis

Statistical differences among samples were estimated using ANOVA (Tukey’s Multiple Comparison Test; GraphPad Prism Version 7.0, Graph Pad Software Inc., San Diego, CA) (fatty acids) and SPSS program (SPSS Inc., Chicago, IL, USA) (carotenoids, volatiles). A value of *p* < 0.05 was considered as statistically significant.

The classification of apricot cultivars was achieved by subjecting the chromatographic data from volatile, fatty acid, and carotenoid profiles to principal component analysis (PCA) with cross validation (full model size and center data). In order to give to all variables included in the analysis an equal chance to influence the model, we used standardization as scaling technique. All the statistical analyses were performed using Unscrambler X software version 10.1 (CAMO Software AS, Oslo, Norway). 

## 3. Results

### 3.1. Carotenoids

Analysis of native (unsaponified) carotenoid extracts is challenging due to their complexity but it has the advantage of revealing the real composition of the samples, which is an important aspect considering the health effects associated with the presence of carotenoids in food [28,29]. Most of the carotenoids in fruits are C40 molecules (tetraterpenes), divided into two major classes: hydrocarbon, or carotenes (acyclic, monocyclic, dicyclic) and oxygenated derivatives, or xanthophylls (carotenols, epoxides, aldehydes or ketones). In many fruits, carotenols are present as free or as mono- or diesters with various fatty acids, generating a wide range of compounds. HPLC-DAD-MS has been proven to be the method of choice for unambiguous identification of carotenoids in complex matrix, exploiting both the specific light absorption properties and the fragmentation pattern of these pigments [13,30]. Tandem mass spectrometry is extremely useful for the analysis of carotenol esters, considering the fact that acylation does not change the chromophore, hence the UV–Vis spectra of parent carotenol overlaps with those of its esters [30]. Here we report the characterization of carotenoids in unsaponified extracts of apricot fruits using HPLC-DAD-APCI-MS, with mass spectra recorded in both positive and negative APCI ionization modes. 

**Figure 1:**
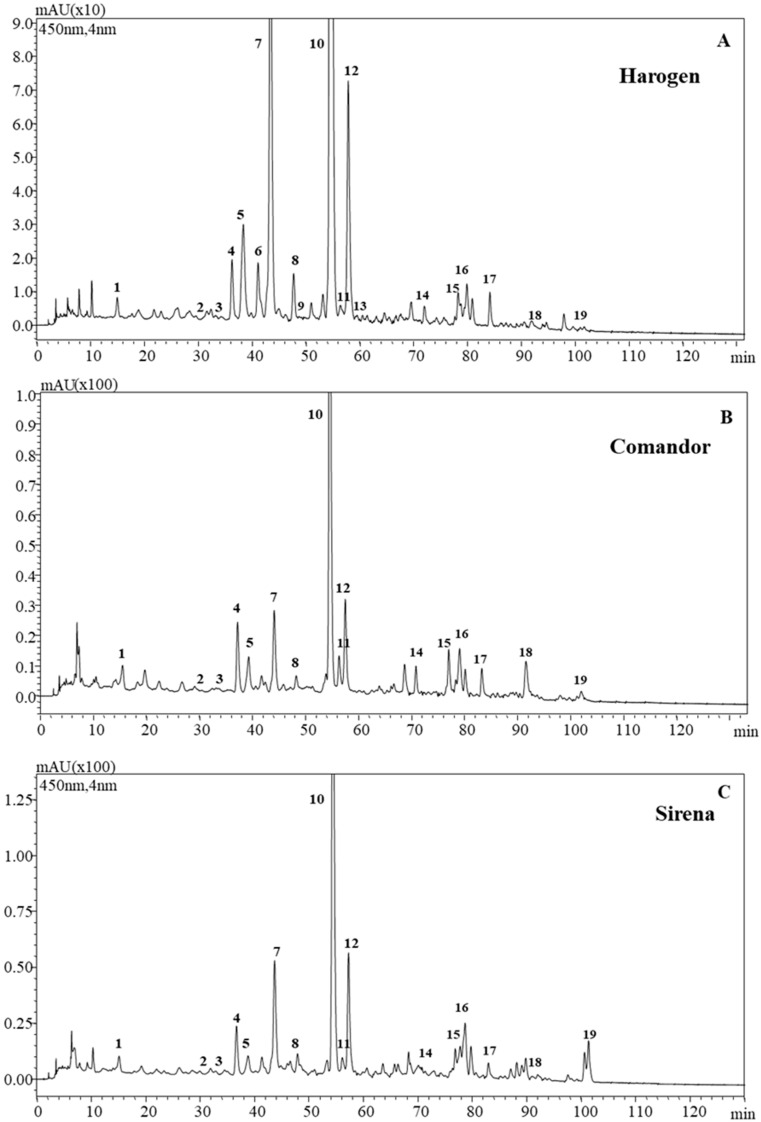
High performance liquid chromatography (HPLC) chromatograms (recorded at 450 nm) of carotenoid extracts from three selected apricot cultivars. (A) Harogem cultivar A1; (B) Comandor cultivar A2; (C) Sirena cultivar A3. For peak assignment see Table 1.

The analyzed apricots cultivars showed a similar native carotenoids pattern, which is illustrated in Figure 1. Figures S1 and S2 (supplementary files) are enlargements of indicated chromatograms, which better show the presence of some peaks. Table 1 reports the UV–Vis and MS APCI (+) and APCI (−) information and identification of the carotenoids in the analyzed apricots cultivars. Nineteen different compounds were detected; from these, β-cryptoxanthin-C18:3 and β-cryptoxanthin-C18:2 were only detected by APCI negative ionization mode, and only in the Harogem cultivar (Figures S3 and S4). The 15-*cis*-β-carotene coeluted with a minor amount of a phytofluene isomer. 

Table 2 shows the carotenoids composition in the 11 cultivars of the analyzed apricots. β-carotene (all*-trans*), 9-*cis*-β-carotene, 13-*cis*-β-Carotene and β-Carotene-5,8 epoxide were the most abundant ones in all samples. “As previously mentioned, except for A4 cultivar, all*-trans*-β-carotene was the major compound in all analyzed samples. With a concentration of β-carotene ranging from 339–2456 µg/100 g FW, the investigated cultivars are comparable with previously reported values. In Hungarian cultivars β-carotene was found at 15 to 39 mg/kg [14]. β-carotene ranged from 585–1075 µg/100 g in apricot cultivars from Croatia [9]. Commercial apricots originating from France and Spain had between 1.44 and 39.97 µg/g β-carotene, as resulted from HPLC analysis on a similar column (C30) with the one used in the present study [17]. In Spanish cultivars β-carotene ranged between 924 and 11058 µg/100 g FW flesh and up to 30500 µg/100 g FW in the peel [7]. Taking into account only the contribution of all*-trans*-β-carotene, most of the cultivars can be considered good to very good sources of pro-vitamin A. As stressed by Ruiz et al., it is important to take into account that unlike other fruits, the peel of apricots (which concentrates higher amounts of carotenoids then the flesh) is consumed as an edible portion. The mono- (9-*cis*; 13-*cis*; 15-*cis*) and di-*cis* isomers (9,13-di-*cis*) of β-carotene have been identified based on their retention times, by comparing their UV–Vis absorption spectra with that of all*-trans-*β-carotene and based on molecular ions (*m/z* 537 (+); 536 (−)) for positive and negative ionization. Specific features of the absorption spectra of carotenoid *cis*-isomers are the hypsochromic (and hypochromic) effect, as well as the appearance of the so-called ”*cis*-peak” in the UV region.

The changes in the absorption spectra are more pronounced when the isomerization occurs near the center of the molecule [31]. C30 columns, which are designed for carotenoid separation, exhibit superior shape selectivity and are currently used for resolution of geometrical isomers [32]. As shown in Table 1, for 9-*cis*-β-carotene, the maximum was shifted to 447 nm (compared to 452 nm for all*-trans* β-carotene) and the *cis*-peak appeared at 345 nm. The presence of *cis*-isomers of β-carotene has been previously reported in Bergeron cultivar [33], in Bergeron, Harogem, Moniqui, Orangered, and Redsun cultivars [17], and in Vitillo cultivar [34]. Even though *cis*-isomers are considered as products of isomerization during the sample preparation or processing, they have been identified as naturally occurring compounds in some fruits, including apricots. *Cis*-isomers of β-carotene (9-*cis* and 15-*cis*) were also found in apricot nectar and they displayed a superior bioaccessibility compared to the all*-trans* form [35]. Moreover, *cis*-isomers (mainly 9-*cis*) of β-carotene can be converted into vitamin A (with 50% conversion rate) and can be beneficial in preventing atherosclerosis and type 2 diabetes [28]. In accordance with Kurtz et al. [17], we could not identify α-carotene in the investigated cultivars, despite the fact that it was previously found in other apricot cultivars [9,15]. Among the other carotenes, γ-carotene (λmax 434, 461, 488; *m/z* 537 (+)) was found in small concentrations in nine of the eleven cultivars analyzed, with the highest concentration in A6 cultivar (195.7 µg/100 g FW) while lycopene was identified in six cultivars, the highest amount being found in A3 cultivar (137 µg/100 g FW). Other studies reported variable concentrations of γ-carotene and/or lycopene [7,14,16,17,34]. We have also detected in small concentrations the colorless carotenoids, phytoene (molecular ion *m/z* 545) and phytofluene (molecular ion *m/z* 543), and specific UV maxima (Table 1). Phytoene and phytofluene reached a maximum of 0.2% in all cultivars. Their presence was previously reported in apricots by Khachick et al. (1989) and Muller (1989), who found 1.05 mg/100 g phytoene and 0.45 mg/100 g phytofluene [16,36]. Recently it was found that apricot commercial juice contains 0.35 mg/100 g phytoene (three isomers) and 0.15 mg/100 g phytofluene (two isomers), and the bioaccessibility of these two carotenoids was superior to most of the other carotenoids in juice [35]. Among the unesterified xanthophylls, we were able to identify β-cryptoxanthin in all the cultivars, ranging from 11.8 (A7) to 137 µg/100 g FW (A2). Lutein was present in 10 of the 11 investigated cultivars, with the highest content in A7 cultivar (129 µg/100 g FW; *p* < 0.05). None of the samples contained zeaxanthin or antheraxanthin. Kurz et al. found lutein in all six cultivars analyzed (0.1–0.36 μg/g FW), zeaxanthin in four cultivars (0.11–0.46 μg/g FW), and β-cryptoxanthin in all cultivars (not quantified) [17]. Fraser and Bramley reported 101 μg/g FW lutein and 31 μg/g FW zeaxanthin. [15]. Lutein was present in two of three cultivars from Croatia (two different locations), while zeaxanthin was only found in one cultivar [9]. In addition to the above-mentioned carotenols, antheraxanthin was identified and quantified in fresh apricot, with an average concentration of 11.5 mg/kg DW [34]. 

As we have analyzed unsaponified extracts, our data are comparable only with those reported by Khachik et al. [16], Breithaupt and Bamedi [37], and Kurz et al. [17], as all the other studies were performed on saponified extracts. Although lutein was present in free form, we could not find lutein mono or diesters in Romanian apricot cultivars. β-Cryptoxanthin was present in esterified form, with six different fatty acids. Considering that fatty acid esters of carotenol have identical absorption spectra with the parent compound, unambiguous identification of carotenol esters is possible only when MS spectra are recorded. The general characteristic ions of carotenol diesters obtained in APCI (positive ionization mode) are: the molecular ion [M+H+ FA1+FA2]^+^, [M+H+FA2]^+^ corresponds to the loss of the first fatty acid esterifying the hydroxyl group; [M+H+FA1]^+^ to the loss of the second fatty acid; [M+H]^+^ to the backbone of the xanthophyll; [FA1]^+^ to the first fatty acid; [FA2]^+^ to the second fatty acid, and respectively to the loss of toluene (*m/z*—92) [30]. In our samples, only monoesters derived from β-cryptoxanthin were identified, laurate (*m/z*—735), palmitate (*m/z*—791), oleate (*m/z*—816), linoleate (*m/z*—814), linolenate (*m/z*—812), and stearate (*m/z*—819) (Table 1). 

It is interesting to note the order of elution, with β-cryptoxanthin-linolenate eluting before all*-trans*-β-carotene, and before the shorter but saturated β-cryptoxanthin-laurate. The presence of methylene interrupted double bonds in polyunsaturated fatty acids (linoleic, linolenic); esterifying the xanthophyll determines an increase of the polarity and a decrease of the retention times on C30 column. In the case of esters with saturated fatty acids, the retention times increased with the carbon chain length of the fatty acid [30].

β-Cryptoxanthin-palmitate was the most common ester, being found in nine of eleven cultivars, with a significantly higher concentration in A10 cultivar (181 µg/100 g FW; *p* < 0.05). It was followed by β-cryptoxanthin-laurate, identified in nine cultivars (lower concentrations) and β-cryptoxanthin-stearate, in five cultivars. The esters of xanthophylls with saturated fatty acids are common constituents in a large variety of fruits and they accumulate in chromoplasts during ripening of fruits [30]. Among the esters with unsaturated fatty acids, the β-cryptoxanthin-oleate was the most abundant, up to 350 µg/100 g FW in A2 cultivar (*p* < 0.05), while linoleate and linolenate were found only in A1 cultivar, in a very low amount. β-Cryptoxanthin and lutein esters with oleic acid were considered as potential markers for authenticity of apricot products as they could not be detected in pumpkin, which can be used for adulteration of apricot jams [17]. Although Khachik et al. [6] analyzed unsaponified extracts from dried and canned apricots using a C18 column and PDA (photodiode array) detector, they did not find carotenol esters. Among the Romanian cultivars, there were also two samples, A4 and A11, which completely lacked esters. Breithaupt and Bamedi, using a C30 column and MS detection with APCI ionization, quantified a total of 458 μg/100 g esters of β-cryptoxanthin and lutein, without specifying the nature of fatty acids [37]. Compared with the work of Kurz et al. [17], we detected, for the first time in apricots, three additional β-cryptoxanthin esters: linoleate, linolenate, and stearate, respectively; but also, β-carotene-5,8 epoxide, and β-zeacarotene. 

The concentration of the identified compounds varied significantly among the investigated apricot cultivars (Table 2). For example, the β-carotene relative percentage ranged from 64% in the A1 cultivar to 23% in the A4 cultivar (*p* < 0.05). Total carotenoid content varied significantly (*p* < 0.05) among the eleven cultivars examined, with the richest cultivar being A11 Tudor (3909.86 μg/100 g FW), followed by Harogem (A1) and Mamaia (A10) cultivars. Sass-Kiss and coworkers [14] revealed a much larger variability of carotenoid composition depending on the apricot cultivar, when compared with sour cherry and tomato. High variation of β-cryptoxanthin content, ranging between 5% in orange flesh apricots and 28% in yellow flesh apricots, was also observed in Spanish cultivars [7]. Analyzing various cultivars with white, yellow, light orange, and orange flesh, the same group reported that the percentages of β-carotene, β-cryptoxanthin, and γ-carotene did not follow any particular pattern. The color of the fruits is an important attribute which strongly influences the perception of the consumers. Previous studies reported that individual and total carotenoid content in both flesh and peel is well correlated with the color parameters (a*, b*, hue angle), and the hue angle being the most appropriate parameter for estimating the carotenoid content in apricots. Interestingly, the authors did not find correlations between the carotenoid content and other parameters, such as maturity stage, flesh firmness, and soluble solids [7].

As the major carotenoids, β-carotene (all*-trans* and *cis* isomers), β-cryptoxanthin (free and esterified), and γ-carotene, have pro-vitamin A activity and fairly good bioavailability; thus, apricots could significantly contribute to the daily intake of retinol. Moreover, it was recently demonstrated that β-cryptoxanthin could contribute to bone health by regulation of bone homeostasis [29]. Although β-cryptoxanthin is mainly found in its esterified form in apricots, previous works showed that the bioavailability of xanthophyll’s esters is comparable to that of the parent compound [30].

### 3.2. Fatty Acids

**Figure 2:**
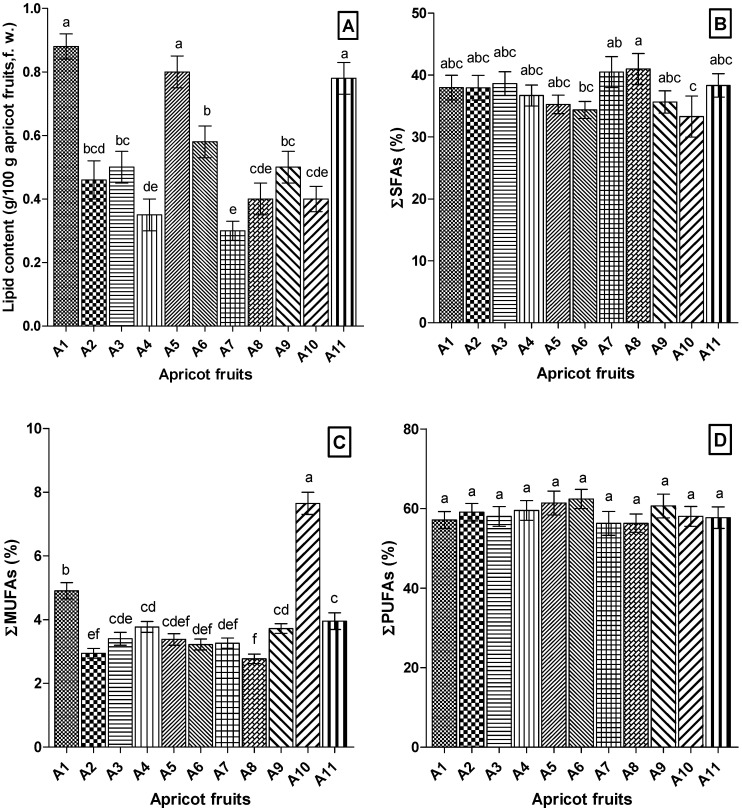
Comparative representation of total lipid contents (**A**) and fatty acid classes of apricot fruit samples: (**B**) SFAs, saturated fatty acids; (**C**) MUFAs, monounsaturated fatty acids; (**D**) PUFAs, polyunsaturated fatty acids. Values are the mean ± SD of three samples, analyzed individually in triplicate (*n* = 3 × 3). Values with different letters (a–f) are significantly different (*p* < 0.05), using ANOVA Tukey’s multiple comparison test. A1–A11, samples of apricots.

Total fruit lipid content varied significantly (*p* < 0.05) among the eleven cultivars examined (Figure 2). The values ranged between 0.30 g/100 g fresh weight (FW) (A7) and 0.88 g/100 g FW (A1). The crude oil contents of many edible fruits are usually lower than 2%, and vary considerably depending on the climate, variety, geographical origin, harvest year, and the methods of cultivation [3,19,38]. 

The physical, chemical, and physiological properties of fats and oils are essentially determined by their fatty acid composition. The fatty acid proportions in apricot fruits are given in Table 3. Seventeen fatty acids were detected in all the samples analyzed. The predominant fatty acids were linoleic (18:2(n-6)), palmitic (16:0), and linolenic (18:3(n-3)) acids with concentrations comprised between 42.09% (A4)–46.99% (A9), 27.27% (A10)–32.70% (A7), and 11.47% (A7)–17.16% (A4), respectively. The research literature indicates that fruit species have specific fatty acid compositions and profiles during development and ripening, although great differences in fatty acid contents among different varieties may also exist within the same species [25,39]. 

The differences in fatty acid amounts are reflected in the total saturated fatty acids (SFAs), total monounsaturated fatty acids (MUFAs), and total polyunsaturated fatty acids (PUFAs) proportions, which are also presented in Figure 2B–D. The concentration of SFAs was about 33.30% of the total FAMEs detected in A10 cultivar, while it was significantly higher (*p* < 0.05) in fruits of cultivar A8 (40.98%). The total lipids of A10 (7.65%) were richer in MUFAs (mainly consisting of oleic acid) than fruit lipids of A8 cultivar (2.77%). It should be noted that the lipids of all the fruit samples analyzed had a remarkable content of PUFAs ranging from 56.28% (A7) to 62.40% (A6). Moreover, all apricot cultivars had fairly good ratios of n-6/n-3 PUFAs, with values ranging from 2.26 (A6) to 3.90 (A7) (Table 1). Several studies suggest that low values of the dietary n-6/n-3 PUFAs ratios (ranging from 1 to 5) can significantly reduce the risk of coronary heart disease, diabetes, and cancer, and can improve autoimmune response [40].

To the best of our knowledge, this is the first work that reports the detailed profile of fatty acids in apricot fruits. Interestingly, numerous papers focused on the lipophilic compounds and fatty acids in apricot kernel oil, a byproduct of fruit processing. Due to the presence of toxic cyanogenic glycosides, the use of apricot kernel oil in food is limited but it can be valuable for cosmetic applications or biodiesel. Compared to the fruits, apricot kernel oil is characterized by a higher content of MUFAs, mainly oleic acid, which represents more than 50% of total fatty acids, followed by linolenic and palmitic acids [23]. 

### 3.3. Volatiles

The volatile aroma compounds from eleven apricot cultivars isolated and analyzed by ITEX/GC-MS technique are grouped in Table 4. More than 300 compounds are reported in literature to have been identified in apricots [41]. Not all of these are important contributors to the specific apricot aroma, their impact depending on the characteristic odor threshold (e.g., some volatiles in low concentration may have a greater impact on the apricot aroma than others that are present in higher concentration) [20]. In the present study, the separated compounds belong to aldehydes (21), esters (24), alcohols (12), ketones (10), terpenes hydrocarbons and oxygenated derivates (27), lactones (3), organic acids (8), and others (5). Terpenoids and esters were the most abundant classes.

In A1, A5, A6, A7, A9, and A11 samples, β-linalool was the main terpenoid, the highest level being retrieved in A6 cultivar (33.52%). This acyclic monoterpene alcohol is a descriptor of floral lavender-like, fresh, citric odor. Linalool was found to be the major terpenoid also in the apricot cultivars analyzed by Xi et al. [42]. Depending on the apricot cultivar, limonene, α-terpineol, β-ionone, and megastigma-4,6(*Z*),8(*E*)-triene were also among the major terpenoids identified. β-ionone is formed by β-carotene degradation contributing with flowery and raspberry notes. Moreover, megastigma-4,6(*Z*),8(*E*)-triene is one of the four isomers (*Z*,*Z*; *E*,*E*; *Z*,*E*; and *E*,*Z*) that are derived from β-ionone via the readily dehydrated β-ionol. These compounds were identified in other fruits, including tropical ones such as starfruits or passion fruit, imparting a rose-like or raspberry-like odor [43]. Other terpenoids that have carotenoids as precursors are geranylacetone (present in all analyzed samples), dihydro-β-ionone, and β-cyclocitral. Xi et al. showed that the light-colored apricot cultivars contained abundant β-ionone and dihydro-β-ionone and a stronger flowery and peach-like aroma, which may suggest that the carotenoid in these cultivars could be cleaved by the specific enzyme into the corresponding apocarotenoids, decisively influencing the color and aroma quality [42]. 

The esters are compounds commonly occurring in fruits and derived foods giving pleasant (floral and fruity) flavor attributes. In the studied apricot samples, 25 esters were tentatively identified, acetates, namely hexyl acetate and butyl acetate being the major ones. These esters were also the main ones found in the apricot cultivars from the Southern Xinjiang region of China, characterized by Feng et al. [41] and in those from Turkey analyzed by Gokbulut et al. [20], being described as contributors to the fruity aroma. Nevertheless, the ester content and profile are related to the botanical origin. Thus, in the 15 cultivar apricots cultivated in Turkey, only relatively low levels of esters were retrieved, the major ones being (*E*)-2-hexenyl propanoate, (*E*)-2- hexenyl butyrate, and hexyl butanoate [20]. In our study, butanoates, hexanoates, and propanoates were found in small percentages. 

Regarding the alcohols group, the main alcohol found in all samples was 1-hexanol (0.29–1.93%). The origin of C_6_ alcohols, such as 1-hexanol, (*E*)-2-hexen-1-ol, and (Z*)*-3-hexen-1-ol, as well as the C_6_ aldehydes (e.g., hexanal) is related to the lipoxygenase activity that is involved in the cleavage of the unsaturated fatty acids [42]. Both C_6_ alcohols and aldehydes are responsible for the herbaceous odor of different fruits, including apricots. Among aldehydes, the predominant ones are hexanal, heptanal, benzaldehyde, octanal, and nonanal; the total aldehydes concentration varies between 9.83–27.04%, depending on the apricot cultivar. 

The richest cultivar in ketones, including lactones, is the A8 cultivar. 6-Methyl-5-hepten-2-one is a degradation compound of lycopene and the principal ketone found in all samples, its level ranging from 1.34% to 9.46%. Together with β-ionone, this ketone is reported to be responsible for the floral notes (sample A8 containing also the highest percentage of β-ionone). The lactones on the other hand are key aroma compound of apricots, imparting a fruity flavor [41,42]. Only three lactones were identified in the analyzed cultivars, with γ-decalactone being the principal one.

### 3.4. PCA Analysis

Principal component analysis (PCA) is one of the most powerful and often used methods for data reduction and exploratory analysis on complex data sets as are those obtained from the exhaustive chemical characterization of vegetable matrices. In the present study, the PCA was applied to all data in order to determine the interrelationships between the eleven selected apricot cultivars allowing us to detect and interpret the sample patterns, their similarities and differences. Thus, the initial data matrix (containing the volatile profiles, the fatty acids, and the carotenoids compositions) is projected onto a smaller number of “latent” variables called principal components (PC), being decomposed by PCA method into multiplication of loading (apricots chemical composition) and score (apricots cultivar samples) matrices. In this way, the quantification of the amount of useful information—as opposed to noise or meaningless variation—is gathered into a model that is easier to interpret than the original data set.

**Figure 3:**
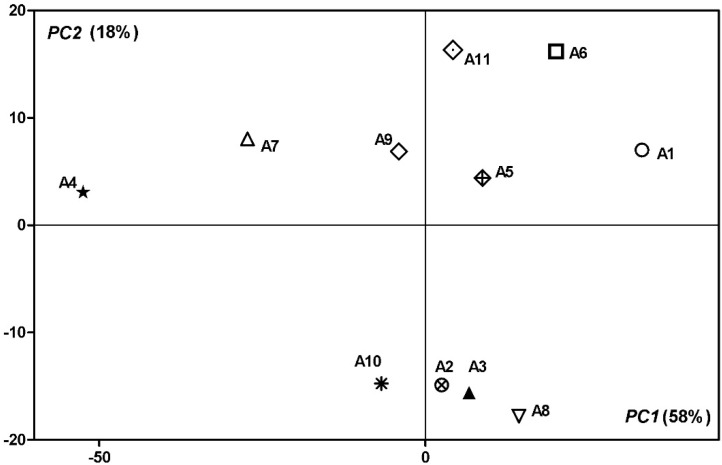
Principal components analysis bi-plots of 11 apricots cultivars based on their carotenoids content, fatty acids, and volatile profiles. The first two components together explain 76% of the data variation.

Thus, by applying this unsupervised method of pattern recognition on all data, the two principal components explained 76% of the overall variance (58% and 18% for PC1 and PC2, respectively) dividing the analyzed cultivars into distinct clusters (Figure 3). In this case, from the correlation loadings, the factors that most contributed to PC1 were the fatty acids 18:1n-9, 15:0, 17:0 and the carotenoids, β-carotene and 13-*cis*-β-carotene. On the other hand, the main contributors to the PC2 were the volatile compounds, namely, *cis*-2-heptenal, butyl acetate, butanoic acid 3-hexenyl ester, gamma-decalactone, and gamma-dodecalactone. As it can be seen in the PCA biplot (Figure 3), the A2, A3, A8, and A10 cultivars have similar volatile profiles.

**Figure 4:**
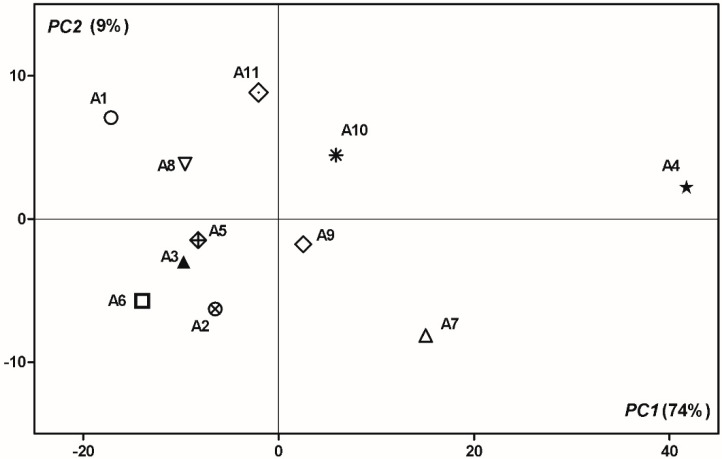
Principal components analysis bi-plots of 11 apricots cultivars based on their carotenoids content and fatty acids profiles. The first two components together explain 83% of the data variation.

If we take into consideration only the fatty acids and carotenoids compositions, 83% of overall variance of data can be explained by the first two principal components (74% and 9% for PC1 and PC2, respectively), showing a better discrimination between the samples (Figure 4). Thus, using PCA, it was revealed that even though the qualitative compositions of the fatty acids and carotenoid compounds of apricot samples are generally similar for all varieties, quantitatively they are specific to each variety, and thus the profile of the fatty acids fraction and the carotenoids profile can be successfully used for variety authentication and discrimination. Compared to the other cultivars, the A4 has a very distinctive profile mainly due to its large amount of β-carotene-5,8 epoxide. In the case of the A7 cultivar, lutein can be considered the marker compound, due to its high content.. From the compounds that can be considered as discrimination markers among apricot varieties, we can mention β-carotene, 9,13-di-*cis*-β-carotene, β-carotene-5,8 epoxide, 15-*cis*-β-carotene, γ-carotene, and the 18:1n-9 fatty acid.

Environmental factors and cultivation practice can affect the accumulation of bioactive compounds in apricots. Dragovic-Uzelak and coworkers compared the polyphenol and carotenoid content in three apricot cultivars grown in two different regions in Croatia [9]. In the case of polyphenols, no significant differences were found depending on the region, while for carotenoids, the cultivars grown in the Mediterranean region had a higher carotenoids content compared to the same cultivars grown in a continental region [9]. Recently, it was found that apricots grown in organic conditions had more than two-fold higher carotenoid content than those obtained from conventional practice [44]. Despite the influence of several other factors, the genotype effect was found as the major factor for the variation in composition of phenolic compounds and carotenoids [5,8,45]. The total carotenoid content in Harogem cultivar in our study (3807.42 ± 194.21 μg/100 g FW) is very close with that previously reported (39.07 ± 2.93 μg/g) for the same cultivar originating from France [17]. Among the Romanian cultivars, Tudor (A11), Mamaia (A10), Comandor (A2), and Olimp (A8) stand as the most recommended with respect to the total carotenoid content. Tudor cultivar also had a good n-6/n-3 fatty acids ratio (3.02), a high content of n-3 PUFAs (14.34%) and a high total lipids content. Due to its location, Dobrogea region (southeast of Romania) offers ideal climate and soil conditions for apricot culture [2]. In order to validate these results, the same cultivars should be tested in different geographic regions and during several years.

## 4. Conclusions

Carotenoids, fatty acids, and volatiles composition was reported here for the first time in nine Romanian cultivars, in comparison with two known international apricot cultivars using chromatographic methods and chemometric analysis. In addition to the already known high β-carotene content, the analysis of unsaponified extracts allowed us to identify β-cryptoxanthin esters in nine of the 11 cultivars, some of them for the first time. With the pro-vitamin A carotenoids, β-carotene, β-cryptoxanthin, γ-carotene, and β-zeacarotene, generally representing more than 80% of the carotenoids, apricots are valuable sources of vitamin A precursors and important contributors to the antioxidant properties of these fruits. Despite the low total lipid content, all apricot cultivars had good n-6/n-3 PUFA ratios which could not only contribute to the overall nutritional value, but also could enhance the bioaccessibility of carotenoids. A very complex volatiles profile was revealed, with compounds derived from degradation of β-carotene and fatty acids. The PCA of data was shown to be a versatile chemometric approach capable of revealing relations between samples’ chemical composition. Thus, the applied PCA highlighted the contribution of the analyzed compound classes to the variability of apricot cultivars composition as well as the specific compounds that could be used as authenticity markers.

## Figures and Tables

**Table 1:** UV-Vis and MS APCI (+) and APCI (−) information and identification of the carotenoids in the analyzed apricots cultivars.

ID	Identification	UV-Vis Maxima	MS Data APCI (+) and (−)
1	Lutein	422, 444, 473	569 (+); 568 (−)
2	Phytoene	s276, 286, s297	545 (+); 544 (−)
3	Phytofluene	333, 347, 367	543 (+); 542 (−)
4	β-Cryptoxanthin	s428, 451, 476	553 (+); 552 (−)
5	β-Carotene-5,8 epoxide	403, 427, 453	553 (+); 552 (−)
6	15-*cis*-β-carotene	337, 420, 449,472	537 (+); 536 (−)
7	13 -*cis*-β-carotene	338, 420, 444, 469	537 (+); 536 (−)
8	9,13-di-*cis*-β-carotene	336, 421, 444, 473	537 (+); 536 (−)
9	β-Cryptoxanthin-C18:3	n.d.	812 (−)
10	All*-trans* β-carotene	421, 452, 478	537 (+); 536 (−)
11	β-Zeacarotene	402, 426, 450	539 (+); 538 (−)
12	9-*cis*-β-carotene	345, 421, 447, 473	537 (+); 536 (−)
13	β-Cryptoxanthin-C18:2	n.d.	814 (−)
14	β-Cryptoxanthin-C12:0	s428, 451, 476	735 (+); 734 (−)
15	β-Cryptoxanthin-C18:1	s428, 451, 476	816 (−)
16	γ-Carotene	434, 461, 488	537 (+); 536 (−)
17	β-Cryptoxanthin-C16:0	s428, 450, 476	791 (+); 790 (−)
18	β-Cryptoxanthin-C18:0	s428, 450, 477	819 (+); 818 (−)
19	Lycopene	448, 471, 503	537 (+); 536 (−)

MS (mass spectrometry); APCI (atmospheric pressure chemical ionization).

**Table 2:** Carotenoids composition (μg/100 g fresh weight (FW) +/− SD^a^) in 11 cultivars of apricots. ^a^ Mean of three replicates, +/− SD (standard deviation). Different superscript letters (^a–h^) in the same row mean significant differences. n.d. = not detected.

ID	Compound	A1	A2	A3	A4	A5	A6	A7	A8	A9	A10	A11
1	Lutein	25.68 ± 1.98^d^	54.41 ± 4.81^b^	34.36 ± 1.98^c^	n.d.	34.99 ± 2.69^c^	18.20 ± 1.56^e^	129.75 ± 9.19^a^	13.95 ± 1.56^ef^	41.25 ± 3.68^c^	1.69 ± 0.23^g^	6.78 ± 0.71^fg^
2	Phytoene	8.56 ± 0.76^a^	3.20 ± 0.34^d^	3.12 ± 0.34^d^	0.50 ± 0.06^e^	0.29 ± 0.06^e^	0.91 ± 0.24^e^	5.90 ± 0.85^b^	3.49 ± 0.41^cd^	3.17 ± 0.38^d^	4.22 ± 0.49^c^	6.38 ± 0.58^b^
3	Phytofluene	4.28 ± 0.42^b^	3.20 ± 0.38^cd^	3.12 ± 0.33^d^	0.97 ± 0.14^e^	0.58 ± 0.14^e^	0.46 ± 0.14^e^	5.90 ± 0.92^a^	3.49 ± 0.49^bcd^	6.35 ± 0.85^a^	4.22 ± 0.42^bc^	6.38 ± 0.71^a^
4	β-Cryptoxanthin	59.92 ± 5.52^d^	137.62 ± 9.62^a^	84.35 ± 6.36^b^	58.14 ± 4.53^d^	46.47 ± 3.11^e^	34.13 ± 1.98^fg^	11.80 ± 0.95^h^	38.35 ± 2.83^ef^	60.29 ± 4.24^cd^	25.30 ± 1.56^g^	70.16 ± 5.52^c^
5	β-Carotene-5,8 epoxide	145.52 ± 9.62^f^	99.21 ± 8.91^f^	40.61 ± 2.97^g^	784.89 ± 70.99^a^	124.90 ± 7.21^f^	34.13 ± 2.26^g^	347.95 ± 18.81^d^	247.55 ± 12.73^e^	257.02 ± 11.46^e^	619.80 ± 31.11^b^	465.58 ± 26.87^c^
6	15-*cis*-β-carotene	77.04 ± 5.94^a^	n.d	n.d.	n.d.	11.62 ± 0.99^e^	63.72 ± 4.10^b^	5.90 ± 0.99^f^	38.35 ± 2.55^c^	6.35 ± 0.55^f^	29.51 ± 1.64^d^	6.38 ± 0.69^f^
7	13 -*cis*-β-carotene	479.36 ± 24.32^b^	179.22 ± 7.50^e^	234.30± 13.72^d^	n.d.	110.37 ± 7.21^f^	65.99 ± 3.82^g^	5.90 ± 0.92^h^	226.63 ± 9.90^d^	114.23 ± 5.66^f^	328.87 ± 15.56^c^	854.63 ± 42.43^a^
8	9,13-di-*cis*-β-carotene	47.08 ± 4.24^c^	19.20 ± 1.70^e^	34.36 ± 1.56^d^	n.d.	8.71 ± 0.85^f^	2.28 ± 0.28^gh^	5.90 ± 0.75^fg^	24.41 ± 1.70^e^	3.17 ± 0.33^fgh^	63.24 ± 4.38^b^	82.91 ± 5.80^a^
9	β-Cryptoxanthin-C18:3	0.63 ± 0.08^a^	n.d.	n.d.	n.d.	n.d.	n.d.	n.d.	n.d	n.d.	n.d.	n.d.
10	all*-trans*-β-carotene	2456.72 ± 110.31^a^	1004.93 ± 39.60^e^	952.82 ± 55.15^e^	339.15 ± 22.91^h^	1002.09 ± 67.88^e^	898.85 ± 55.15^ef^	536.67 ± 24.47^g^	1541.08 ± 66.47^c^	786.91 ± 41.01^f^	1277.54 ± 56.57^d^	1766.65 ± 77.78^b^
11	β-Zeacarotene	17.12 ± 1.27^e^	54.41 ± 4.53^d^	37.49 ± 2.97^d^	7.75 ± 0.52^ef^	66.81 ± 6.08^b^	100.12 ± 5.94^a^	64.87 ± 4.53^b^	94.14 ± 7.07^a^	60.29 ± 5.37^bc^	67.46 ± 5.09^b^	n.d.
12	9-*cis*-β-carotene	269.64 ± 14.14^cd^	169.62 ± 10.61^g^	231.18 ± 11.46^ef^	300.39 ± 17.54^bc^	246.89 ± 15.56^de^	93.30 ± 5.52^h^	318.47 ± 16.97^b^	198.74 ± 9.90^fg^	298.27 ± 12.73^bc^	274.06 ± 13.86^cd^	644.16 ± 36.77^a^
13	β-Cryptoxanthin-C18:2	1.89 ± 0.16^a^	n.d.	n.d.	n.d.	n.d.	n.d.	n.d.	n.d.	n.d.	n.d.	n.d.
14	β-Cryptoxanthin-C12:0	27.76 ± 2.05^f^	136.21 ± 9.76^a^	14.63 ± 0.98^g^	n.d.	46.36 ± 3.25^e^	45.40 ± 3.25^e^	19.61 ± 1.88f^g^	102.02 ± 6.22^b^	60.50 ± 5.80^d^	74.11 ± 5.23^c^	n.d.
15	β-Cryptoxanthin-C18:1	44.34 ± 3.54^d^	350.51 ± 16. 97^a^	27.35 ± 1.98^e^	n.d.	n.d.	n.d.	n.d.	n.d.	157.09 ± 8.49^c^	187.42 ± 11.03^b^	n.d.
16	γ-Carotene	77.04 ± 5. 37^d^	169.62 ± 11.31^b^	155.09 ± 8.91^b^	n.d.	106.48 ± 7.07^d^	195.70± 15.56^a^	88.46 ± 6.51^d^	83.68 ± 7.07^d^	22.21 ± 1.84^f^	54.81 ± 4.24^e^	n.d.
17	β-Cryptoxanthin-C16:0	42.84 ± 2.83^f^	137.30 ± 10. 18^b^	114.99 ± 6.08^c^	n.d.	91.38 ± 5.52^d^	73.22 ± 4.38^e^	21.08 ± 2.12^g^	79.77 ± 5.66^de^	79.51 ± 5.66^de^	181.09 ± 12.73^a^	n.d.
18	β-Cryptoxanthin-C18:0	21.57 ± 1.56^d^	311.85 ± 15.56^a^	19.22 ± 1.41^d^	n.d.	n.d.	n.d.	n.d.	128.86 ± 7.06^b^	n.d.	51.06 ± 3.96^c^	n.d.
19	Lycopene	0.43 ± 0.10^d^	3.20 ± 0.30^d^	137.06± 11.60^a^	n.d.	13.31 ± 2.26^c^	36.41 ± 3.82^b^	n.d	6.97 ± 0.78^cd^	0.74 ± 0.14^d^	n.d.	n.d.
	**Total carotenoids**	3807.42 ± 194.21^a^	2833.70 ± 152.06^c^	2124.06 ± 127.79^d^	1491.79 ± 116.69^f^	1911.25 ± 129.88^de^	1662.81 ± 108.00^ef^	1568.15 ± 89.84^f^	2831.47 ± 142.40^c^	1957.34 ± 108.17^de^	3244.40 ± 168.11^b^	3909.86 ± 197.85^a^

**Table 3:** Fatty acid composition (% of total fatty acids) of total lipids in fruits of eleven apricot cultivars *.

Fatty Acids	A1	A2	A3	A4	A5	A6	A7	A8	A9	A10	A11
8:0	0.02^c^	0.02^c^	0.05^abc^	0.03^bc^	0.03^bc^	0.08^a^	0.06^ab^	0.04^bc^	0.08^a^	0.02^c^	0.04^bc^
10:0	0.04^bc^	0.03^bcd^	0.03^bcd^	0.01^d^	0.02^bcd^	0.09^a^	0.05^b^	0.02^bcd^	0.01^cd^	0.03^bcd^	0.10^a^
12:0	0.24^f^	0.19^g^	0.24^f^	0.40^b^	0.24^f^	0.54^a^	0.28^de^	0.22^fg^	0.30^d^	0.25^ef^	0.33^cd^
14:0	0.30^d^	0.28^d^	0.35^c^	0.08^e^	0.27^d^	0.35^c^	0.53^a^	0.27^d^	0.27^d^	0.27^d^	0.40^b^
15:0	0.20^a^	0.12^e^	0.16^bc^	0.17^bc^	0.18^ab^	0.11^e^	0.15^cd^	0.13^de^	0.13^de^	0.20^a^	0.12^e^
16:0	29.29^abc^	31.13^ab^	32.69^a^	30.90^ab^	29.30^abc^	28.69^bc^	32.70^a^	30.12^abc^	30.38^abc^	27.27^c^	30.44^abc^
16:1(n-9)	0.21^ab^	0.15^de^	0.19^b^	0.23^a^	0.15^de^	0.19^b^	0.15^de^	0.16^cd^	0.20^b^	0.13^e^	0.14^de^
16:1(n-7)	0.18^de^	0.08^h^	0.17^ef^	0.24^b^	0.21^c^	0.20^cd^	0.12^g^	0.15^f^	0.18^de^	0.32^a^	0.17^ef^
16:2(n-4)	0.18^c^	0.07^h^	0.13d^e^	0.26^a^	0.04^i^	0.12^e^	0.13^e^	0.10^fg^	0.10^g^	0.22^b^	0.01^j^
17:0	0.42^b^	0.21^f^	0.32^cd^	0.46^a^	0.31^cd^	0.15^g^	0.26^e^	0.34^c^	0.20^f^	0.30^d^	0.29^de^
18:0	3.28^de^	3.97^ab^	3.04^efg^	3.13^ef^	3.20^ef^	2.83^fg^	4.15^a^	3.78^abc^	2.67^g^	3.68^bcd^	3.41^cde^
18:1(n-9)	1.53^cd^	1.51^cd^	1.36^d^	1.40^cd^	1.41^cd^	1.32^d^	1.61^c^	1.43^cd^	1.36^d^	4.64^a^	2.38^b^
18:1(n-7)	1.73^cd^	1.20^gh^	1.68^de^	1.90^bc^	1.61^de^	1.51^ef^	1.38^fg^	1.03^h^	1.98^b^	2.57^a^	1.28^g^
18:2(n-6)	46.53^a^	46.16^a^	43.57^a^	42.09^a^	44.68^a^	43.17^a^	44.68^a^	42.79^a^	46.99^a^	45.66^a^	43.37^a^
18:3(n-3)	14.26^c^	12.90^cde^	14.30^c^	17.16^b^	16.66^b^	19.11^a^	11.47^e^	13.40^cd^	13.55^cd^	12.15^de^	14.34^c^
20:0	1.09^abc^	1.02^bcd^	0.97^cde^	0.89^ef^	1.10^ab^	0.84^f^	1.13^ab^	1.21^a^	0.97^cde^	0.96^def^	1.04^bcd^
22:0	0.52^h^	0.97^ef^	0.77^fg^	0.64^gh^	0.60^gh^	0.69^gh^	1.14^de^	4.81^a^	0.64^gh^	1.33^cd^	2.15^b^
*∑n-3 PUFAs*	14.26^c^	12.90^cde^	14.30^c^	17.16^b^	16.66^b^	19.11^a^	11.47^e^	13.40^cd^	13.55^cd^	12.15^de^	14.34^c^
*∑n-6 PUFAs*	46.53^a^	46.16 ^a^	43.57 ^a^	42.09 ^a^	44.68 ^a^	43.17 ^a^	44.68 ^a^	42.79 ^a^	46.99 ^a^	45.66 ^a^	43.37 ^a^
*∑n-9 PUFAs*	1.74 ^c^	1.66 ^c^	1.55 ^c^	1.63 ^c^	1.56 ^c^	1.51 ^c^	1.76^c^	1.59 ^c^	1.56 ^c^	4.77^a^	2.52^b^
*n-6 / n-3*	3.26^cde^	3.58^abc^	3.05^ef^	2.45^gh^	2.68^fg^	2.26^h^	3.90^a^	3.19^de^	3.47^bcd^	3.76^ab^	3.02^ef^

* The values represent the means of three samples, analyzed individually in triplicate (*n* = 3 × 3). Different superscript letters (^a–j^) in the same row mean significant differences (*p* < 0.05), using ANOVA Tukey’s multiple comparison test. A1–A11, samples of apricot. Polyunsaturated fatty acids (PUFAs); caprylic acid (8:0); capric acid (10:0); lauric acid (12:0); myristic acid (14:0); pentadecanoic acid (15:0); palmitic acid (16:0); *cis*-7 hexadecenoic acid (16:1(n-9)); palmitoleic acid (16:1(n-7)); 9,12-hexadecadienoic acid (16:2(*n*-4)); margaric acid (17:0); stearic acid (18:0); oleic acid (18:1(n-9)); vaccenic acid (18:1(n-7)); linoleic acid (18:2(n-6)); α- linolenic acid (18:3(n-3)); arachidic acid (20:0); behenic acid (22:0); tr. = trace.

**Table 4:** Composition of volatiles in the 11 apricot cultivars *.

Compound	A1	A2	A3	A4	A5	A6	A7	A8	A9	A10	A11
***Alcohols***											
1-Pentanol	0.24 ± 0.07^d^	0.54 ± 0.06^a^	0.35 ± 0.02^c^	0.13 ± 0.03^e^	0.22 ± 0.08^d^	0.21 ± 0.03^d^	0.23 ± 0.05^d^	0.41 ± 0.16^b^	0.35 ± 0.11^c^	0.41 ± 0.04^b^	0.26 ± 0.06^d^
3-Pentanol	n.d.	n.d.	n.d.	0.13 ± 0.04^b^	0.14 ± 0.06^b^	n.d.	0.16 ± 0.04^a^	n.d.	n.d.	n.d.	n.d.
3-Hexen-1-ol, (*Z*)-	0.50 ± 0.01^b^	n.d.	0.67 ± 0.13^a^	0.13 ± 0.01^f^	0.33 ± 0.05^d^	0.51 ± 0.05^b^	0.25 ± 0.04^e^	0.40 ± 0.05^c^	0.72 ± 0.13^a^	n.d.	n.d.
2-Hexen-1-ol, (*E*)-	0.32 ± 0.07^a^	n.d.	n.d.	n.d.	n.d.	n.d.	n.d.	n.d.	n.d.	n.d.	n.d.
1-Hexanol	1.93 ± 0.30^a^	1.00 ± 0.28^d^	1.51 ± 0.24^b^	0.43 ± 0.01^fg^	0.32 ± 0.01^g^	0.52 ± 0.11^f^	0.30 ± 0.11^g^	0.70 ± 0.01^e^	1.05 ± 0.16^d^	0.29 ± 0.01^g^	1.29 ± 0.30^c^
Phenol	0.21 ± 0.06^d^	0.64 ± 0.22^b^	n.d.	0.51 ± 0.11^c^	0.71 ± 0.07^a^	n.d.	0.49 ± 0.11^c^	n.d.	n.d.	0.76 ± 0.17^a^	n.d.
1-Hexanol, 2-ethyl-	0.18 ± 0.01^c^	n.d.	0.16 ± 0.02^c^	0.04 ± 0.01^f^	0.11 ± 0.02^d^	0.16 ± 0.01^c^	0.11 ± 0.02^d^	0.16 ± 0.02^c^	0.31 ± 0.12^a^	0.25 ± 0.04^b^	0.07 ± 0.04^e^
1-Octanol	0.06 ± 0.01^d^	n.d.	n.d.	0.13 ± 0.03^c^	0.29 ± 0.08^a^	n.d.	0.19 ± 0.06^b^	n.d.	n.d.	n.d.	n.d.
1-Nonanol	n.d.	n.d.	n.d.	n.d.	0.12 ± 0.01^a^	n.d.	n.d.	n.d.	n.d.	n.d.	n.d.
1-Decanol	n.d.	n.d.	n.d.	n.d.	0.24 ± 0.04^a^	n.d.	n.d.	n.d.	n.d.	n.d.	n.d.
Phenol, 2-(1,1-dimethylethyl)-4-methyl-	n.d.	1.03 ± 0.04^d^	1.61 ± 0.32^b^	0.91 ± 0.10^d^	n.d.	0.52 ± 0.04^e^	n.d.	2.74 ± 0.13^a^	1.27 ± 0.17^c^	1.38 ± 0.21^c^	n.d.
1-Heptadecanol	n.d.	0.43 ± 0.09^b^	n.d.	1.29 ± 0.16^a^	n.d.	0.21 ± 0.02^d^	n.d.	0.22 ± 0.01^d^	n.d.	0.31 ± 0.04^c^	n.d.
**Total**	3.44 ± 0.53^b^	3.64 ± 0.69^b^	4.3 ± 0.73^a^	3.70 ± 0.50^b^	2.48 ± 0.42^c^	2.13 ± 0.26^cd^	1.73 ± 0.43^de^	4.63 ± 0.38^a^	3.70 ± 0.69^b^	3.4 ± 0.51^b^	1.62 ± 0.40e
***Aldehydes***											
Hexanal	8.06 ± 0.11^c^	5.81 ± 1.00^de^	9.13 ± 0.02^bc^	3.46 ± 0.18^f^	8.86 ± 0.45^bc^	5.12 ± 0.18^e^	6.38 ± 0.3^d^	9.41 ± 0.74^b^	5.86 ± 0.28^de^	16.15 ± 1.12^a^	9.70 ± 0.07^b^
Furfural	n.d.	2.68 ± 0.18^a^	n.d.	n.d.	n.d.	0.15 ± 0.04^b^	n.d.	n.d.	n.d.	n.d.	n.d.
2-Hexenal, (*E*)-	0.51 ± 0.09^d^	0.5 ± 0.06^d^	1.49 ± 0.22^b^	0.37 ± 0.05^d^	1.43 ± 0.47^b^	1.05 ± 0.09^c^	0.96 ± 0.06^c^	0.42 ± 0.13^d^	n.d.	2.63 ± 0.52^a^	0.53 ± 0.27^d^
4-Heptenal, (*Z*)-	0.16 ± 0.07^f^	0.27 ± 0.08^d^	0.51 ± 0.05^a^	0.16 ± 0.01^f^	0.30 ± 0.11^cd^	0.33 ± 0.11^bc^	0.21 ± 0.04^e^	0.37 ± 0.05^b^	n.d.	n.d.	0.26 ± 0.03^d^
Heptanal	1.42 ± 0.05^cd^	2.30 ± 0.28^a^	2.53 ± 0.21^a^	0.93 ± 0.01^e^	1.67 ± 0.15^bc^	1.88 ± 0.15^b^	1.38 ± 0.08^d^	2.45 ± 0.33^a^	1.07 ± 0.19^e^	2.54 ± 0.13^a^	1.94 ± 0.08^b^
2-Heptenal, (*Z*)-	n.d.	0.53 ± 0.18^c^	0.48 ± 0.01^c^	0.22 ± 0.01^e^	n.d.	0.30 ± 0.04^d^	0.28 ± 0.1^d^	0.60 ± 0.03^b^	n.d.	0.67 ± 0.06^a^	0.21 ± 0.01^e^
Benzaldehyde	2.48 ± 0.18^a^	1.86 ± 0.11^c^	1.43 ± 0.15^de^	0.83 ± 0.16^h^	1.80 ± 0.30^c^	0.92 ± 0.07^gh^	1.29 ± 0.07^ef^	1.53 ± 0.27^d^	1.32 ± 0.20^def^	2.15 ± 0.27^b^	1.12 ± 0.16^fg^
Octanal	0.96 ± 0.15^d^	1.64 ± 0.06^b^	1.27 ± 0.10^c^	0.93 ± 0.11^d^	1.67 ± 0.40^b^	1.19 ± 0.22^c^	1.26 ± 0.09^c^	1.69 ± 0.06^b^	1.20 ± 0.12^c^	2.07 ± 0.10^a^	0.76 ± 0.23^d^
Benzeneacetaldehyde	n.d.	0.32 ± 0.17^a^	n.d.	0.06 ± 0.01^b^	n.d.	n.d.	0.05 ± 0.01^b^	n.d.	n.d.	n.d.	n.d.
2-Octenal, (*E*)-	0.26 ± 0.06^e^	0.71 ± 0.06^c^	0.86 ± 0.06^b^	0.30 ± 0.01^e^	0.41 ± 0.05^d^	0.69 ± 0.13^c^	0.43 ± 0.08^d^	1.03 ± 0.11^a^	n.d.	0.72 ± 0.04^c^	0.48 ± 0.01^d^
2,5-Furandicarboxaldehyde	n.d.	1.00 ± 0.10^a^	n.d.	n.d.	n.d.	n.d.	n.d.	n.d.	n.d.	n.d.	n.d.
Nonanal	0.87 ± 0.19^e^	1.57 ± 0.18^b^	1.14 ± 0.28^cd^	0.89 ± 0.08^e^	1.32 ± 0.30^c^	1.20 ± 0.23^c^	1.66 ± 0.22^b^	1.57 ± 0.39^b^	0.95 ± 0.06^de^	2.57 ± 0.06^a^	0.80 ± 0.24^e^
5-Hydroxymethylfurfural	n.d.	4.46 ± 0.28^a^	n.d.	n.d.	0.39 ± 0.08^d^	1.37 ± 0.27^b^	n.d.	n.d.	0.95 ± 0.40^c^	n.d.	0.32 ± 0.45^d^
Decanal	0.37 ± 0.10^f^	1.06 ± 0.15^b^	0.65 ± 0.14^d^	0.50 ± 0.06^e^	1.07 ± 0.08^b^	0.68 ± 0.06^d^	0.88 ± 0.08^c^	0.88 ± 0.04^c^	0.58 ± 0.08^de^	1.37 ± 0.10^a^	0.32 ± 0.11^f^
*trans*-2-Nonenal	n.d.	n.d.	n.d.	n.d.	n.d.	n.d.	0.22 ± 0.01^a^	n.d.	n.d.	n.d.	n.d.
*trans*-2-Decenal	n.d.	n.d.	n.d.	0.12 ± 0.01^c^	0.28 ± 0.08^a^	0.03 ± 0.02^d^	n.d.	n.d.	n.d.	0.16 ± 0.02^b^	n.d.
2-Undecenal	n.d.	0.56 ± 0.06^a^	n.d.	0.27 ± 0.04^c^	0.27 ± 0.06^c^	0.18 ± 0.01^d^	0.38 ± 0.06^b^	n.d.	n.d.	n.d.	n.d.
Octadecanal	n.d.	0.80 ± 0.21^a^	n.d.	n.d.	n.d.	n.d.	n.d.	n.d.	n.d.	n.d.	n.d.
Tetradecanal	n.d.	0.57 ± 0.11^a^	n.d.	0.49 ± 0.04^b^	0.13 ± 0.05^e^	0.19 ± 0.01^d^	0.50 ± 0.08^b^	0.30 ± 0.04^c^	0.10 ± 0.01^e^	0.50 ± 0.17^b^	n.d.
Dodecanal	n.d.	n.d.	n.d.	n.d.	n.d.	0.46 ± 0.08^b^	0.40 ± 0.03^c^	n.d.	n.d.	0.66 ± 0.18^a^	n.d.
Tridecanal	n.d.	0.40 ± 0.02^b^	0.26 ± 0.06^cd^	0.3 ± 0.07^c^	0.24 ± 0.03^d^	0.16 ± 0.01^e^	0.40 ± 0.04^b^	0.41 ± 0.11^b^	n.d.	0.71 ± 0.10^a^	n.d.
**Total**	15.09 ± 1.00^d^	27.04 ± 3.29^b^	19.75 ± 1.30^c^	9.83 ± 0.85^e^	19.84 ± 2.61^c^	15.9 ± 1.72^d^	16.68 ± 1.35^d^	20.66 ± 2.30^c^	12.03 ± 1.34^e^	32.90 ± 2.87^a^	16.44 ± 1.64^d^
***Ketones***											
2-Hexanone	n.d.	n.d.	n.d.	n.d.	0.26 ± 0.07^a^	0.10 ± 0.01^c^	n.d.	n.d.	n.d.	n.d.	0.13 ± 0.09^b^
2-Heptanone	n.d.	n.d.	n.d.	0.06 ± 0.01^b^	n.d.	n.d.	n.d.	0.19 ± 0.04^a^	n.d.	n.d.	n.d.
2-Methyl-6-heptanone	0.21 ± 0.01^c^	n.d.	n.d.	0.09 ± 0.02^e^	n.d.	0.10 ± 0.03^e^	0.16 ± 0.03^d^	0.21 ± 0.05^c^	n.d.	0.31 ± 0.06^a^	0.28 ± 0.01^b^
6-methyl-5-Hepten-2-one	2.92 ± 0.01^e^	1.89 ± 0.25^f^	4.85 ± 0.29^c^	1.34 ± 0.08^f^	3.65 ± 0.25^d^	1.95 ± 0.08^f^	4.69 ± 0.47^c^	9.46 ± 0.25^a^	4.49 ± 0.49^c^	7.33 ± 0.35^b^	3.35 ± 0.04^de^
2,5-Hexanedione	n.d.	n.d.	0.18 ± 0.04^b^	n.d.	n.d.	0.12 ± 0.03^bc^	3.09 ± 0.32^a^	0.15 ± 0.06^b^	n.d.	0.12 ± 0.02^bc^	n.d.
1,1,3-Trimethyl-2-cyclohexanone	0.77 ± 0.01^b^	n.d.	n.d.	0.07 ± 0.03^f^	n.d.	0.13 ± 0.03^e^	0.08 ± 0.04^ef^	0.52 ± 0.01^c^	n.d.	0.28 ± 0.10^d^	0.85 ± 0.02^a^
2-Cyclohexen-1-one, 3-methyl-	1.18 ± 0.07^a^	n.d.	n.d.	n.d.	n.d.	n.d.	n.d.	n.d.	0.75 ± 0.16^b^	0.29 ± 0.08^d^	0.63 ± 0.13^c^
α-Isophorone	0.42 ± 0.05^bc^	n.d.	n.d.	n.d.	n.d.	0.32 ± 0.04^c^	n.d.	0.39 ± 0.06^b^	n.d.	n.d.	0.45 ± 0.17^a^
Acetophenone	0.68 ± 0.16^e^	1.19 ± 0.10^b^	0.84 ± 0.25^d^	0.6 ± 0.13^e^	1.14 ± 0.06^b^	0.56 ± 0.12^e^	0.87 ± 0.08^cd^	0.95 ± 0.07^cd^	0.99 ± 0.13^c^	1.37 ± 0.22^a^	0.59 ± 0.04^e^
m-Methylacetophenone	0.22 ± 0.10^a^	n.d.	n.d.	0.10 ± 0.01^c^	n.d.	0.17 ± 0.04^b^	n.d.	n.d.	n.d.	n.d.	0.17 ± 0.06^b^
**Total**	6.40 ± 0.41^d^	3.08 ± 0.35^fg^	5.87 ± 0.58^de^	2.26 ± 0.28^g^	5.05 ± 0.38^e^	3.45 ± 0.38^f^	8.89 ± 0.94^b^	11.87 ± 0.54^a^	6.23 ± 0.78^d^	9.70 ± 0.83^b^	6.45 ± 0.55^d^
***Esters***											
Acetic acid, 2-methylpropyl ester	0.22 ± 0.01^g^	0.77 ± 0.13^bc^	0.87 ± 0.36^b^	0.26 ± 0.06^fg^	0.78 ± 0.21^bc^	0.72 ± 0.06^c^	0.49 ± 0.09^d^	0.83 ± 0.06^b^	0.37 ± 0.16^e^	1.41 ± 0.04^a^	0.36 ± 0.07^ef^
Butanoic acid, 2-methyl-, methyl ester	n.d.	n.d.	n.d.	n.d.	n.d.	n.d.	n.d.	n.d.	n.d.	0.12 ± 0.02^a^	n.d.
Butanoic acid, ethyl ester	1.07 ± 0.05^d^	2.73 ± 0.13^a^	1.01 ± 0.18^d^	1.31 ± 0.28^c^	0.90 ± 0.13^de^	0.75 ± 0.11^e^	1.01 ± 0.41^d^	1.43 ± 0.40^bc^	1.04 ± 0.3^d^	1.51 ± 0.35^b^	0.98 ± 0.28^d^
*n*-Butyl acetate	4.60 ± 0.16^e^	7.75 ± 0.40^c^	14.42 ± 3.29^a^	4.9 ± 0.51^e^	7.15 ± 0.72^c^	8.04 ± 0.27^c^	5.65 ± 0.67^de^	13.69 ± 1.22^a^	7.65 ± 0.29^c^	11.48 ± 0.84^b^	6.89 ± 0.53^cd^
Butanoic acid, 2-oxo-, methyl ester	n.d.	0.42 ± 0.19^cd^	0.30 ± 0.13^e^	0.48 ± 0.04^bc^	0.79 ± 0.19^a^	0.16 ± 0.01^f^	0.51 ± 0.06^b^	0.33 ± 0.01^e^	n.d.	0.39 ± 0.18^d^	n.d.
Butanoic acid, 2-methyl-, ethyl ester	n.d.	0.68 ± 0.21^a^	n.d.	0.15 ± 0.02^b^	n.d.	n.d.	n.d.	0.12 ± 0.04^c^	n.d.	n.d.	0.08 ± 0.01^d^
2-Propenoic acid, butyl ester	n.d.	n.d.	n.d.	n.d.	n.d.	0.06 ± 0.01^a^	n.d.	n.d.	n.d.	n.d.	n.d.
Acetic acid, pentyl ester	n.d.	n.d.	0.15 ± 0.04^a^	0.1 ± 0.02^b^	n.d.	n.d.	n.d.	n.d.	n.d.	n.d.	n.d.
Hexanoic acid, methyl ester	n.d.	n.d.	n.d.	n.d.	n.d.	0.10 ± 0.02^a^	n.d.	n.d.	n.d.	n.d.	n.d.
Butanoic acid, butyl ester	0.30 ± 0.03^c^	n.d.	0.50 ± 0.36^b^	n.d.	n.d.	0.14 ± 0.04^d^	n.d.	n.d.	1.11 ± 0.02^a^	n.d.	0.11 ± 0.15^d^
Hexanoic acid, ethyl ester	0.40 ± 0.08^cd^	4.48 ± 0.77^a^	0.37 ± 0.01^cde^	0.76 ± 0.06^b^	n.d.	0.15 ± 0.01^fg^	n.d.	0.48 ± 0.01^c^	n.d.	0.24 ± 0.13^def^	0.20 ± 0.09^ef^
Acetic acid cis-3-hexenyl ester	1.14 ± 0.18^d^	1.05 ± 0.16^d^	2.51 ± 0.17^b^	1.07 ± 0.06^d^	0.07 ± 0.02^f^	0.13 ± 0.01^ef^	2.96 ± 0.09^a^	0.34 ± 0.04^e^	2.27 ± 0.43^c^	2.05 ± 0.21^c^	0.08 ± 0.02^f^
Acetic acid, hexyl ester	4.01 ± 0.01^d^	8.03 ± 0.88^b^	11.72 ± 0.25^a^	4.25 ± 0.15^cd^	0.07 ± 0.01^f^	0.35 ± 0.13f	2.20 ± 0.03^e^	0.72 ± 0.11^f^	4.88 ± 0.86^c^	2.49 ± 0.37^e^	1.78 ± 0.45^e^
2-Hexen-1-ol, acetate, (*E*)-	0.73 ± 0.12^d^	1.59 ± 0.16^b^	1.20 ± 0.08^c^	4.17 ± 0.62^a^	0.06 ± 0.01^e^	n.d.	0.69 ± 0.08^d^	0.60 ± 0.01^d^	1.17 ± 0.47^c^	1.07 ± 0.19^c^	0.16 ± 0.01^e^
Propanoic acid, 2-methyl-, 3-methylbutyl ester	n.d.	n.d.	n.d.	n.d.	n.d.	0.11 ± 0.02^a^	n.d.	n.d.	n.d.	n.d.	n.d.
Propanoic acid, 2-methyl-, 3-hexenyl ester, (*Z*)	n.d.	n.d.	n.d.	n.d.	n.d.	n.d.	0.47 ± 0.04^a^	n.d.	n.d.	n.d.	n.d.
Propanoic acid, 2-methyl-, hexyl ester	n.d.	n.d.	n.d.	n.d.	n.d.	n.d.	0.38 ± 0.06^a^	n.d.	n.d.	n.d.	n.d.
Butanoic acid, 2-hexenyl ester, (*E*)-	n.d.	n.d.	n.d.	n.d.	n.d.	n.d.	0.26 ± 0.07^b^	n.d.	0.35 ± 0.10^a^	n.d.	n.d.
Butanoic acid, 3-hexenyl ester, (*Z*)-	n.d.	n.d.	n.d.	n.d.	0.42 ± 0.04^b^	0.43 ± 0.02^b^	0.11 ± 0.01^c^	n.d.	0.48 ± 0.13^a^	n.d.	n.d.
Butanoic acid, hexyl ester	n.d.	n.d.	2.81 ± 0.91^a^	n.d.	n.d.	0.16 ± 0.06^b^	n.d.	n.d.	n.d.	n.d.	n.d.
Butanoic acid, 3-methyl-, 3-hexenyl ester, (*Z*)-	n.d.	n.d.	n.d.	n.d.	n.d.	0.13 ± 0.05^b^	0.95 ± 0.17^a^	n.d.	n.d.	n.d.	n.d.
Hexanoic acid, 3-hexenyl ester, (*Z*)-	n.d.	n.d.	n.d.	n.d.	n.d.	0.09 ± 0.03^a^	n.d.	n.d.	n.d.	n.d.	n.d.
Hexanoic acid, hexyl ester	n.d.	n.d.	n.d.	n.d.	n.d.	0.10 ± 0.01^a^	n.d.	n.d.	n.d.	n.d.	n.d.
1,2-Benzenedicarboxylic acid, bis(2-methylpropyl) ester	0.22 ± 0.04^e^	n.d.	0.60 ± 0.16^bc^	n.d.	2.46 ± 0.15^a^	0.50 ± 0.12^c^	n.d.	0.67 ± 0.13^b^	0.34 ± 0.11^d^	n.d.	0.21 ± 0.09^e^
**Total**	12.69 ± 0.67^f^	27.50 ± 3.03^b^	36.46 ± 5.94^a^	17.45 ± 1.82^de^	12.70 ± 1.48^f^	12.12 ± 0.97^f^	15.68 ± 1.77^e^	19.21 ± 2.02^cd^	19.66 ± 2.87^cd^	20.76 ± 2.33^c^	10.85 ± 1.71^f^
***Terpenoids***											
α-Pinene	0.16 ± 0.01^gh^	0.68 ± 0.25^a^	0.29 ± 0.06^cd^	0.14 ± 0.02^g^	0.20 ± 0.04^fg^	0.18 ± 0.06^fgh^	0.26 ± 0.06^de^	0.35 ± 0.16^b^	0.20 ± 0.09^fg^	0.33 ± 0.11^bc^	0.22 ± 0.11^ef^
β-*trans*-Ocimene	0.51 ± 0.21^b^	n.d.	n.d.	0.06 ± 0.03^e^	0.16 ± 0.01^d^	0.60 ± 0.07^a^	n.d.	n.d.	n.d.	n.d.	0.45 ± 0.06^c^
β-cis-Ocimene	2.17 ± 0.39^a^	n.d.	n.d.	0.47 ± 0.08^e^	0.72 ± 0.08^d^	1.94 ± 0.08^b^	0.45 ± 0.01e	n.d.	1.56 ± 0.29^c^	n.d.	1.71 ± 0.33^c^
β-Pinene	0.23 ± 0.09^d^	n.d.	n.d.	0.16 ± 0.02^e^	n.d.	0.27 ± 0.06^c^	0.36 ± 0.06^b^	0.54 ± 0.14^a^	0.40 ± 0.16^b^	0.26 ± 0.10^cd^	0.15 ± 0.03^e^
β-Myrcene	2.95 ± 0.09^c^	1.57 ± 0.07^ef^	1.73 ± 0.30^ef^	1.13 ± 0.14^g^	1.43 ± 0.29^fg^	2.41 ± 0.26^d^	1.86 ± 0.13^e^	n.d.	3.30 ± 0.41^b^	2.81 ± 0.21^c^	3.75 ± 0.03^a^
α-Terpinolene	1.12 ± 0.08^a^	n.d.	n.d.	n.d.	n.d.	n.d.	n.d.	n.d.	n.d.	n.d.	n.d.
2-Carene	1.69 ± 0.22^d^	2.30 ± 0.02^bc^	2.56 ± 0.36^b^	0.84 ± 0.11^f^	1.16 ± 0.10^e^	1.64 ± 0.22^d^	1.48 ± 0.05^d^	2.79 ± 0.85^a^	2.50 ± 0.47^b^	2.14 ± 0.44^c^	1.59 ± 0.42^d^
*p*-Cymene	0.60 ± 0.04^a^	n.d.	0.27 ± 0.08^cd^	0.19 ± 0.01^fg^	0.15 ± 0.06^g^	0.32 ± 0.03^c^	0.26 ± 0.05^de^	0.20 ± 0.02^fg^	0.65 ± 0.01^a^	0.21 ± 0.01^ef^	0.52 ± 0.06^b^
d-Limonene	4.01 ± 0.11^a^	1.54 ± 0.11^cd^	1.39 ± 0.55^d^	1.36 ± 0.04^d^	1.37 ± 0.23^d^	3.34 ± 0.16^b^	1.68 ± 0.20^cd^	1.47 ± 0.23^cd^	4.13 ± 0.14^a^	1.77 ± 0.15^c^	3.80 ± 0.21^a^
(+)-4-Carene	1.98 ± 0.40^a^	n.d.	n.d.	0.40 ± 0.07^e^	0.64 ± 0.04^d^	1.66 ± 0.17^b^	0.21 ± 0.12^f^	n.d.	0.52 ± 0.28^de^	n.d.	0.98 ± 0.02^c^
*p*-Cymenene	1.46 ± 0.21^a^	n.d.	n.d.	0.31 ± 0.11^d^	0.29 ± 0.08^d^	0.90 ± 0.11^c^	0.28 ± 0.06^d^	n.d.	1.44 ± 0.25^a^	n.d.	1.32 ± 0.26^b^
Benzene, 1-methoxy-4-(1-methylethyl)-	1.36 ± 0.14^a^	n.d.	n.d.	n.d.	n.d.	n.d.	n.d.	n.d.	n.d.	0.91 ± 0.18^b^	1.34 ± 0.25^a^
β-Linalool	25.97 ± 0.25^b^	2.89 ± 0.19^f^	2.71 ± 0.30^f^	9.06 ± 0.40^e^	20.33 ± 0.92^c^	33.52 ± 1.28^a^	16.45 ± 1.21^d^	2.53 ± 0.33^f^	22 ± 1.45^c^	2.01 ± 0.30^f^	31.55 ± 1.20^a^
p-Cymen-8-ol	0.10 ± 0.02^b^	n.d.	n.d.	n.d.	n.d.	n.d.	n.d.	n.d.	n.d.	n.d.	0.12 ± 0.05^a^
α-Terpineol	4.00 ± 0.02^b^	1.13 ± 0.01^d^	n.d.	n.d.	3.10 ± 0.02^c^	4.76 ± 0.42^a^	n.d.	1.13 ± 0.10^d^	n.d.	n.d.	4.47 ± 0.06^a^
1,3,8-p-Menthatriene	0.39 ± 0.02^b^	n.d.	n.d.	n.d.	n.d.	0.45 ± 0.06^a^	n.d.	n.d.	n.d.	n.d.	0.34 ± 0.04^c^
β-Cyclocitral	1.66 ± 0.14^a^	n.d.	n.d.	n.d.	0.42 ± 0.11^d^	n.d.	0.38 ± 0.06^d^	0.60 ± 0.11^c^	n.d.	1.00 ± 0.11^b^	1.67 ± 0.07^a^
cis-Geraniol	1.45 ± 0.34^bc^	n.d.	n.d.	0.44 ± 0.09^e^	1.18 ± 0.02^d^	1.39 ± 0.38^c^	0.48 ± 0.12^e^	n.d.	1.60 ± 0.34^b^	n.d.	2.02 ± 0.03^a^
α-Citral	0.19 ± 0.11^a^	n.d.	n.d.	n.d.	n.d.	n.d.	n.d.	n.d.	n.d.	n.d.	0.13 ± 0.03^b^
Geranylacetone	0.96 ± 0.14^e^	0.83 ± 0.17^ef^	2.61 ± 0.13^b^	0.63 ± 0.05^f^	1.96 ± 0.18^c^	0.61 ± 0.03^f^	2.19 ± 0.07^c^	3.71 ± 0.36^a^	0.81 ± 0.02^ef^	2.65 ± 0.30^b^	1.38 ± 0.12^d^
β-Ionone	0.94 ± 0.19^f^	4.09 ± 0.16^c^	2.68 ± 0.12^d^	6.84 ± 0.91^b^	n.d.	1.35 ± 0.18^ef^	1.47 ± 0.19^ef^	9.23 ± 0.04^a^	n.d.	1.76 ± 0.21^e^	1.04 ± 0.21^f^
Dihydro-β-ionone	n.d.	0.80 ± 0.15^c^	1.71 ± 0.17^a^	1.44 ± 0.05^b^	0.08 ± 0.01^e^	0.33 ± 0.03^d^	n.d.	1.62 ± 0.12^a^	0.33 ± 0.12^d^	n.d.	n.d.
β-Ionol	n.d.	n.d.	2.11 ± 0.11^a^	0.52 ± 0.11^d^	0.69 ± 0.03^c^	n.d.	n.d.	1.09 ± 0.41^b^	n.d.	n.d.	n.d.
Farnesyl acetone	0.06 ± 0.01^d^	n.d.	0.58 ± 0.01^b^	n.d.	n.d.	n.d.	n.d.	0.86 ± 0.15^a^	n.d.	n.d.	0.20 ± 0.01^c^
Linalool 3,7-oxide	0.70 ± 0.16^c^	n.d.	n.d.	0.24 ± 0.06^e^	0.48 ± 0.11^d^	0.96 ± 0.09^a^	0.17 ± 0.03^e^	n.d.	0.81 ± 0.13^b^	n.d.	0.67 ± 0.15^c^
3-Caranol	n.d.	n.d.	n.d.	1.21 ± 0.02^c^	n.d.	n.d.	1.83 ± 0.28^b^	n.d.	6.52 ± 0.55^a^	n.d.	n.d.
Megastigma-4,6(*Z*),8(*E*)-triene	n.d.	3.06 ± 0.35^c^	4.18 ± 0.23^b^	3.10 ± 0.26^c^	1.33 ± 0.30^e^	1.84 ± 0.20^d^	n.d.	6.39 ± 0.31^a^	2.73 ± 0.23^c^	n.d.	n.d.
**Total**	54.66 ± 3.39^ab^	18.89 ± 1.48^fg^	22.82 ± 2.42^ef^	28.54 ± 2.58^de^	35.69 ± 2.63^c^	58.47 ± 3.89^a^	29.81 ± 2.7^cd^	32.51 ± 3.33^cd^	49.5 ± 4.94^b^	15.85 ± 2.11^g^	59.42 ± 3.75^a^
***Lactones***											
Butyrolactone	0.60 ± 0.17^b^	n.d.	n.d.	n.d.	n.d.	n.d.	n.d.	n.d.	1.43 ± 0.29^a^	n.d.	n.d.
γ-Dodecalactone	0.22 ± 0.05^ef^	1.01 ± 0.04^a^	0.81 ± 0.06^c^	n.d.	n.d.	n.d.	n.d.	0.63 ± 0.07^d^	0.29 ± 0.05^e^	0.89 ± 0.07^b^	0.21 ± 0.01^f^
γ-Decalactone	0.29 ± 0.08^f^	1.20 ± 0.13^a^	0.90 ± 0.14^b^	0.32 ± 0.01^f^	0.73 ± 0.08^c^	0.30 ± 0.06^f^	n.d.	0.89 ± 0.05^b^	0.43 ± 0.04^e^	0.56 ± 0.21^d^	0.36 ± 0.03^ef^
**Total**	1.11 ± 0.30^d^	2.21 ± 0.17^a^	1.71 ± 0.20^b^	0.32 ± 0.01^f^	0.73 ± 0.08^e^	0.30 ± 0.06^f^	n.d.	1.52 ± 0.12^c^	2.15 ± 0.38^a^	1.45 ± 0.28^c^	0.57 ± 0.04^e^
***Acids***											
Butanoic acid, 3-methyl-	0.56 ± 0.16^a^	n.d.	0.34 ± 0.07^b^	0.10 ± 0.02^d^	n.d.	0.20 ± 0.02^c^	0.11 ± 0.02^d^	0.21 ± 0.01^c^	n.d.	n.d.	n.d.
Benzoic Acid	0.28 ± 0.03^b^	n.d.	n.d.	n.d.	0.52 ± 0.06^a^	n.d.	n.d.	n.d.	n.d.	n.d.	n.d.
Octanoic Acid	n.d.	n.d.	n.d.	n.d.	0.32 ± 0.03^a^	n.d.	n.d.	n.d.	n.d.	n.d.	n.d.
Nonanoic acid	n.d.	n.d.	n.d.	n.d.	0.58 ± 0.06^a^	n.d.	0.06 ± 0.01^b^	n.d.	n.d.	n.d.	n.d.
n-Decanoic acid	n.d.	n.d.	n.d.	n.d.	0.29 ± 0.04^a^	n.d.	n.d.	n.d.	n.d.	n.d.	n.d.
Dodecanoic acid	n.d.	1.75 ± 0.17^d^	n.d.	7.43 ± 1.29^a^	4.43 ± 0.16^b^	0.35 ± 0.08^e^	2.44 ± 0.64^c^	0.44 ± 0.08^e^	n.d.	1.67 ± 0.39^d^	n.d.
Tetradecanoic acid	n.d.	4.68 ± 0.95^d^	0.71 ± 0.13^ef^	17.72 ± 0.54^a^	6.50 ± 0.04^c^	1.53 ± 0.21^e^	9.27 ± 1.53^b^	1.49 ± 0.28^e^	n.d.	3.96 ± 0.21^d^	n.d.
Pentadecanoic acid	0.06 ± 0.01^g^	3.89 ± 0.59^d^	1.13 ± 0.25^f^	9.60 ± 1.15^b^	2.91 ± 0.15^e^	1.12 ± 0.02^f^	11.39 ± 1.36^a^	1.47 ± 0.26^f^	n.d.	4.95 ± 0.25^c^	0.13 ± 0.08^g^
**Total**	0.90 ± 0.20^fg^	10.32 ± 1.71^d^	2.18 ± 0.45^ef^	34.85 ± 3.00^a^	15.55 ± 0.54^c^	3.20 ± 0.33^e^	23.27 ± 3.56^b^	3.61 ± 0.62^e^	n.d.	10.58 ± 0.85^d^	0.13 ± 0.08^fg^
***Others***											
Benzene, 2-methyl-1,4-bis(1-methylethyl)-	n.d.	0.42 ± 0.13^c^	0.54 ± 0.15^b^	0.49 ± 0.08^bc^	n.d.	n.d.	n.d.	1.26 ± 0.07^a^	0.34 ± 0.23^d^	n.d.	n.d.
Naphthalene, 1-methyl-	0.22 ± 0.09^de^	n.d.	n.d.	0.19 ± 0.08^ef^	0.52 ± 0.03^bc^	0.22 ± 0.01^de^	0.29 ± 0.06^d^	0.58 ± 0.09^b^	1.27 ± 0.19^a^	0.46 ± 0.02^c^	0.14 ± 0.01^f^
Naphthalene, 2-methyl-	n.d.	n.d.	n.d.	n.d.	n.d.	n.d.	n.d.	n.d.	0.49 ± 0.15^a^	n.d.	n.d.
2,4,6-Octatriene, 2,6-dimethyl-, (*E*,*Z*)-	1.08 ± 0.08^a^	n.d.	n.d.	0.33 ± 0.05^d^	n.d.	n.d.	0.25 ± 0.11^e^	n.d.	0.82 ± 0.22^c^	n.d.	0.94 ± 0.18^b^
Cyclobutene, bis(1-methylethylidene)-	0.39 ± 0.01^b^	n.d.	n.d.	0.11 ± 0.03^d^	n.d.	0.47 ± 0.06^a^	n.d.	n.d.	0.33 ± 0.05^c^	n.d.	0.35 ± 0.13^c^
**Total**	1.69 ± 0.18^b^	0.42 ± 0.13^f^	0.54 ± 0.15^ef^	1.12 ± 0.24^d^	0.52 ± 0.03^ef^	0.69 ± 0.07^e^	0.54 ± 0.17^ef^	1.84 ± 0.16^b^	3.25 ± 0.84^a^	0.46 ± 0.02^f^	1.43 ± 0.32^c^

* Mean % composition of three replicates, +/− SD (standard deviation). Different superscript letters (^a–h^) in the same row mean significant differences. n.d. = not detected.

## Supplementary Materials

The following are available online at https://www.mdpi.com/2076-3921/9/7/562/s1, Figure S1: Enlargements from the chromatogram of the Harogem cultivar at (A) λ = 286 nm, which shows well the peak 2 (phytoene), and (B) at λ = 347 nm which shows well the peak 3 (phytofluene); Figure S2: Enlargments from corresponding chromatograms of other cultivars: Comandor (A) and Sirena (B) at λ = 450 nm, which shows a better resolution and higher contents of peaks 15, 18, and 19; Figure S3: Extracted ion chromatogram of β-cryptoxanthin-C18:3 at m/z 812, APCI(^−^) (A) and β-cryptoxanthin-C18:3 APCI(^−^) MS spectrum (B); Figure S4: Extracted ion chromatogram of β-cryptoxanthin-C18:2 at m/z 814, APCI(^−^) (A) and β-cryptoxanthin-C18:2 at *m/z* 814, APCI-MS spectrum (B).
