# Supplementary figures and images for: Carotenoids, Fatty Acids, and Volatile Compounds in Apricot Cultivars from Romania—A Chemometric Approach
Source: Antioxidants (Basel). 2020 Jun 27;9(7):562. doi: 10.3390/antiox9070562 (PMC7402126; doi:10.3390/antiox9070562)

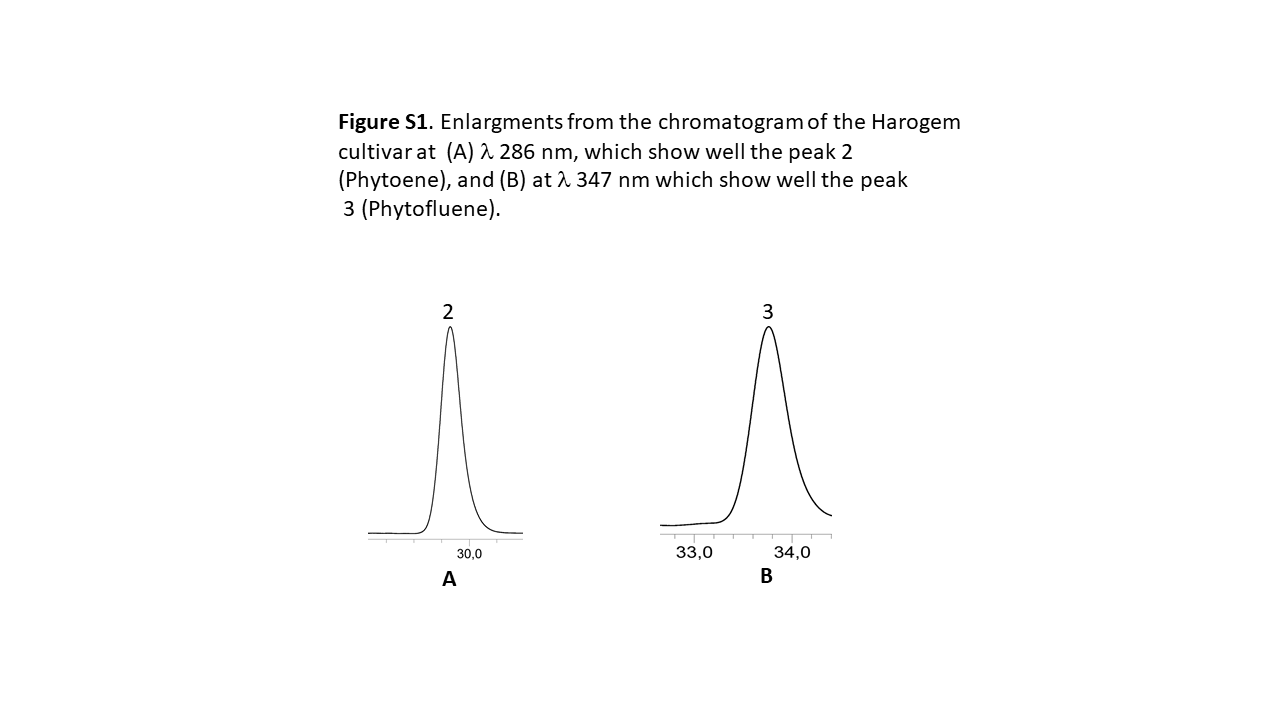

Supplement: Supplementary file 1 [file antioxidants-09-00562-s001.zip › Supplementary files_TIFF/Figure S1.tif]

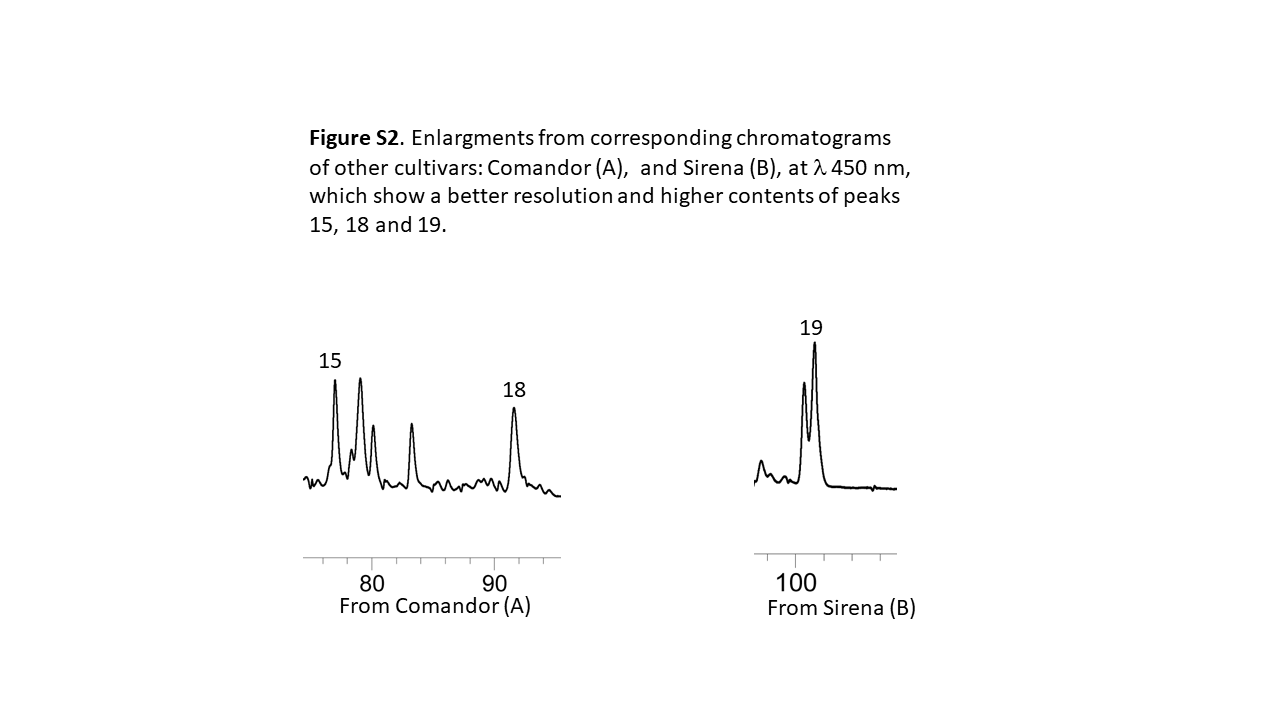

Supplement: Supplementary file 1 [file antioxidants-09-00562-s001.zip › Supplementary files_TIFF/Figure S2.tif]

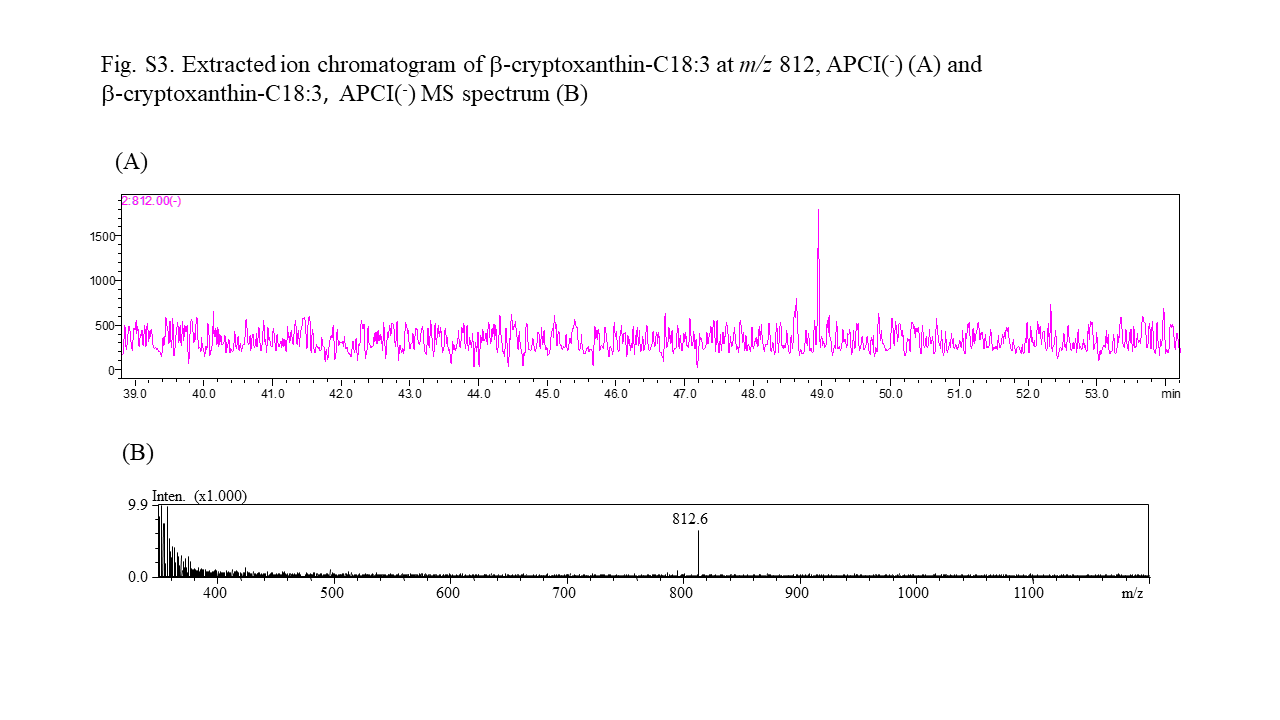

Supplement: Supplementary file 1 [file antioxidants-09-00562-s001.zip › Supplementary files_TIFF/Figure S3.tif]

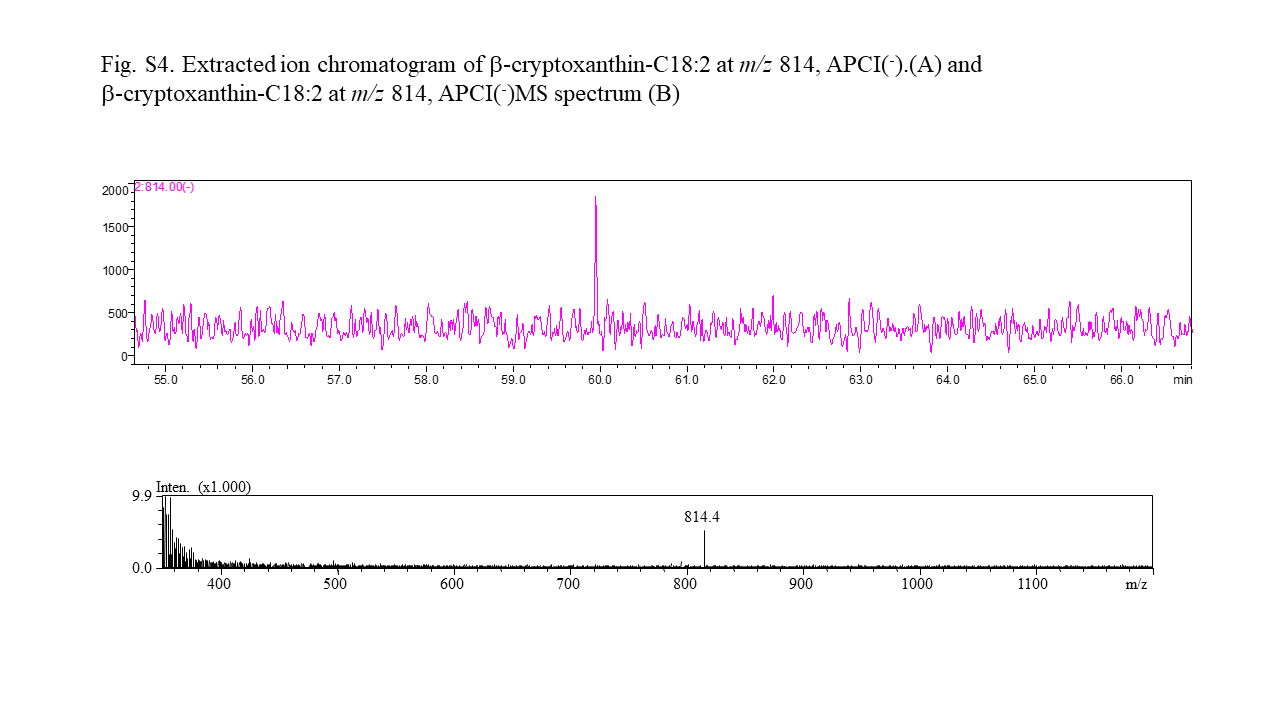

Supplement: Supplementary file 1 [file antioxidants-09-00562-s001.zip › Supplementary files_TIFF/Figure S4.tif]
